# Metal-free synthesis of 3-trifluoromethyl-1,2,4-triazoles *via* multi-component reaction of trifluoroacetimidoyl chlorides, hydrazine hydrate and benzene-1,3,5-triyl triformate

**DOI:** 10.3389/fchem.2022.1013977

**Published:** 2022-09-20

**Authors:** Binjie Wang, Yue Sun, An Cheng, Yeanlun Zhu, Jiye Wang, Zhengkai Chen, Xiao-Feng Wu

**Affiliations:** ^1^ Key Laboratory of Drug Prevention and Control Technology of Zhejiang Province, The Department of Criminal Science and Technology, Zhejiang Police College, Hangzhou, China; ^2^ Key Laboratory of Surface & Interface Science of Polymer Materials of Zhejiang Province, Department of Chemistry, Zhejiang Sci-Tech University, Hangzhou, China; ^3^ Dalian National Laboratory for Clean Energy, Dalian Institute of Chemical Physics, Chinese Academy of Sciences, Dalian, Liaoning, China; ^4^ Leibniz-Institut für Katalyse e. V, Rostock, Germany

**Keywords:** metal-free, multi-component reaction, trifluoromethyl-1,2,4-triazole, trifluoroacetimidoyl chloride, benzene-1,3,5-triyl triformate

## Abstract

A convenient approach for the construction of pharmaceutically valuable 3-trifluoromethyl-1,2,4-triazoles has been developed, which employs the readily available trifluoroacetimidoyl chlorides, hydrazine hydrate and benzene-1,3,5-triyl triformate (TFBen) as starting materials. The multi-component reaction features broad substrate scope, high efficiency, and scalability, providing a facile and straightforward route to the biologically important 3-trifluoromethyl-1,2,4-triazole scaffolds in moderate to good yields. Considering its broad-spectrum pharmaceutical activity, the method offers the opportunity for the further study towards the toxicity risk assessment and structure-activity relationship of the pharmaceuticals containing trifluoromethyl-1,2,4-triazole cores.

## Introduction

**FIGURE 1:**
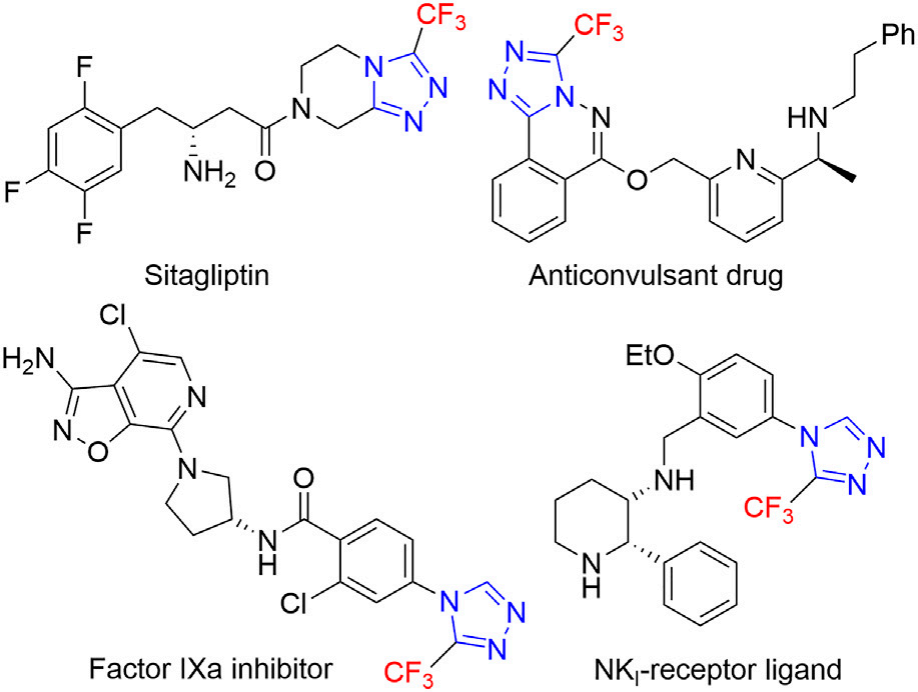
Selected examples of bioactive molecules containing 1,2,4-triazole cores.

1,2,4-Triazoles, especially trifluoromethyl-substituted 1,2,4-triazoles, have found extensive applications in the field of pharmaceutical, agrochemicals, biology, functional materials, and ligand chemistry (Koltin and Hitchcock, 1997; Shivarama Holla, et al., 2002; Haycock-Lewandowski, et al., 2008; Tao, et al., 2010; Zhou and Wang, 2012; Romagnoli, et al., 2014). For instance, the commercial sitagliptin is a potent inhibitor of DPP-IV and is used as a new treatment for type II diabetes (Hansen, et al., 2005). Other trifluoromethyl-1,2,4-triazole derivatives, have been applied as anticonvulsant drug, GlyT1 inhibitor, anti-HIV-1 reagent, and NKI-receptor ligand (Figure 1) (Lebsack, et al., 2004; Girardet, et al., 2006; Syvanen, et al., 2007; Sugane, et al., 2011; Sakurada, et al., 2015). It is well-known that the occurrence of trifluoromethyl group could significantly improve the physicochemical and pharmacological properties of the parent molecules due to the unique character of fluorine atom (Müller, et al., 2007; Gillis, et al., 2015; Zhou, et al., 2016; Han, et al., 2020). Therefore, the exploration of efficient and practical strategies for the preparation of trifluoromethyl-1,2,4-triazoles is highly desirable.

**FIGURE 2:**
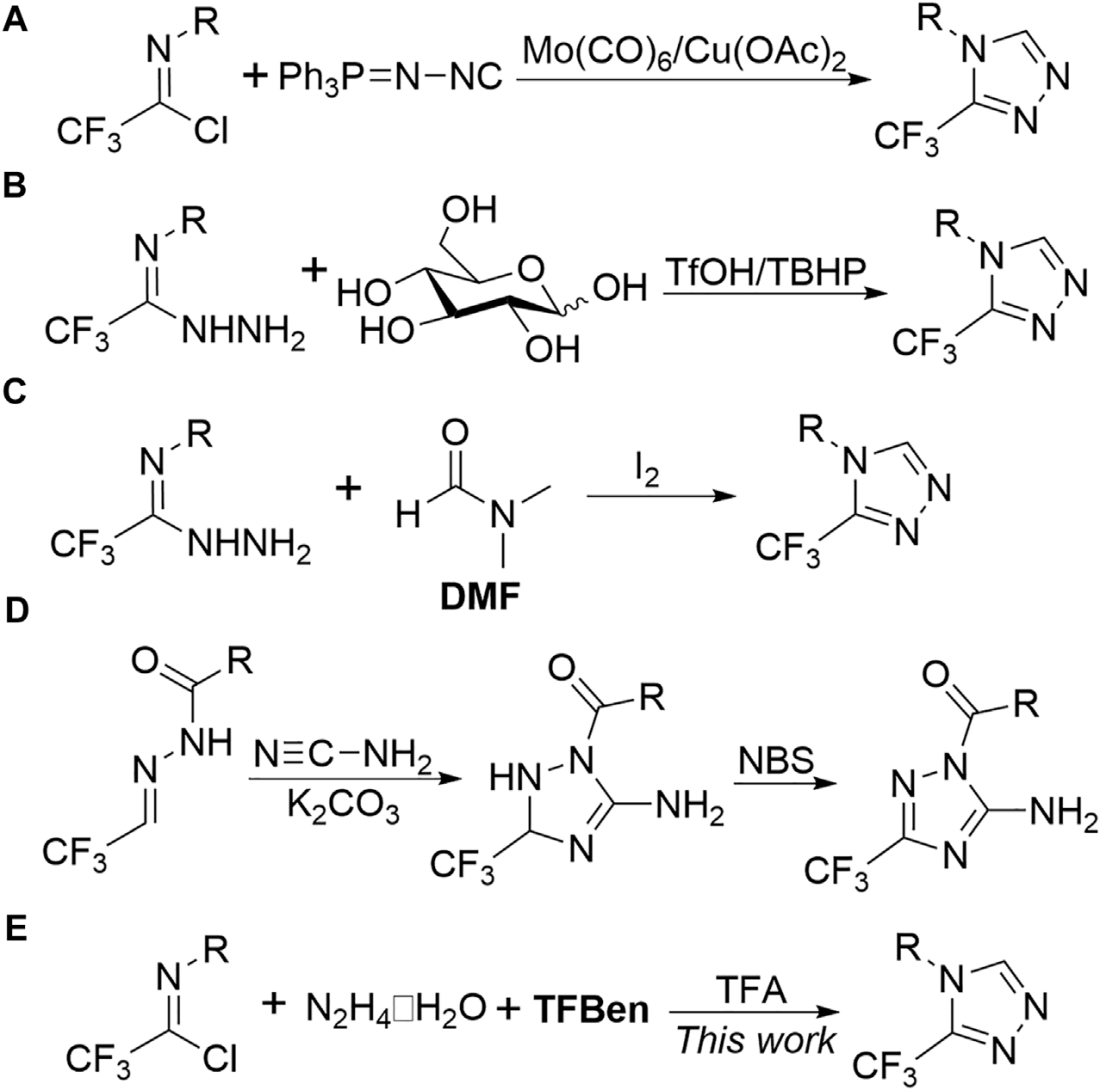
Several approaches for the synthesis of 3-trifluoromethyl-1,2,4-triazoles.

Traditional methods for the synthesis of trifluoromethyl-1,2,4-triazoles usually suffer from tedious reaction procedures, narrow substrate scope and lower efficiency (Buscemi, et al., 2003; Buscemi, et al., 2006; Funabiki, et al., 1999; Sibgatulin, et al., 2010). Recent years have witnessed considerable achievements about the construction of trifluoromethyl-substituted 1,2,4-triazoles (Zhang, et al., 2019), which include transition metal-catalyzed three-component reaction of aryldiazonium salts with fluorinated diazo reagents and nitriles (Peng, et al., 2020). Our groups also developed a series of convenient approaches for the assembly of this kind of important five-membered *N*-heterocycle by using trifluoroacetimidoyl chlorides (Hu, et al., 2019; Du, et al., 2020) and trifluoroacetimidohydrazides (Zhang, et al., 2021a; Zhang, et al., 2021b; Zhang, et al., 2021c; Lu, et al., 2022a; Zhang, et al., 2022) as versatile trifluoromethyl synthons. Compared with the in-depth study toward the synthesis of 5-trifluoromethyl-1,2,4-triazoles, the relevant reports regarding the formation of the more specific 3-trifluoromethyl-1,2,4-triazoles have been rare but still of great significance. Wu, Chen and co-workers reported a copper-mediated [3 + 2] cycloaddition of trifluoroacetimidoyl chlorides and *N*-isocyanoiminotriphenylphphorane (NIITP) to efficiently access 3-trifluoromethyl-1,2,4-triazoles (Figure 2A) (Yang, et al., 2021). They also utilized D-glucose (Lu, et al., 2021) and *N,N*-dimethylformamide (DMF) (Lu, et al., 2022b) as an inexpensive C1 source to realize [4 + 1] cyclization reaction with trifluoroacetimidohydrazides for preparing 3-trifluoromethyl-1,2,4-triazoles (Figures 2B,C). Very recently, Hu and co-workers described a tandem addition/cyclization reaction of trifluoromethyl *N*-acylhydrazones and cyanamide to afford polysubstituted 3-trifluoromethyl-1,2,4-triazolines, which could be oxidized to 1,2,4-triazoles with NBS (Figure 2D) (Liu, et al., 2022). Despite notable advances having been gained, other facile pathways to access the valuable trifluoromethyl-substituted *N*-heterocycles deserve to be further investigated.

Benzene-1,3,5-triyl triformate (TFBen) is first designed and developed by Wu and co-workers and has usually been used as a potent CO surrogate in diverse carbonylative transformations (Jiang, et al., 2016; Yang, et al., 2022). Meanwhile, TFBen is also adopted as a C1 source in the formation of a variety of heterocycles. Wu group reported a metal-free annulation of hydrazides with benzene-1,3,5-triyl triformate (TFBen) to produce 1,3,4-oxadiazoles (Yin, et al., 2018). Our group disclosed a palladium-catalyzed three-component carbonylative reaction of trifluoroacetimidohydrazides and aryl iodides for delivering 5-trifluoromethyl-1,2,4-triazoles (Tang, et al., 2021). In these reactions, TFBen provided a CO unit to form carbonyl-containing compounds and the latter underwent an intramolecular dehydration process. In continuation of our effort on the carbonylative reaction using CO surrogate for the efficient construction of trifluoromethyl-containing heterocycles (Chen, et al., 2020a; Chen, et al., 2020b; Yang, et al., 2020; Tang, et al., 2021; Wang, et al., 2021), we herein presented a multi-component annulation reaction of readily available trifluoroacetimidoyl chlorides (Tamura, et al., 1993; Chen, et al., 2020c), hydrazine hydrate and benzene-1,3,5-triyl triformate for the metal-free synthesis of 3-trifluoromethyl-1,2,4-triazoles (Figure 2E).

## Results and discussion

The study was initiated by the employment of trifluoroacetimidoyl chloride **1e** as model substrate along with hydrazine hydrate and benzene-1,3,5-triyl triformate (TFBen) as starting materials (Table 1). The reaction proceeded smoothly in the presence of TsOH H_2_O in toluene at 100°C for 12 h, and the desired 3-trifluoromethyl-1,2,4-triazole product **3e** was isolated in 53% yield (Table 1, entry 1). Other acidic additives were also examined, including TfOH, PivOH and TFA, and the results indicated that TFA promoted the reaction with highest efficiency (Table 1, entries 2–4). Then, a series of organic solvents were tried to test the solvent effect of this reaction. The multi-component reaction could occur in various solvents, but the obtained reaction yields were all inferior to that of toluene (Table 1, entries 5–9). Lowering and elevating the reaction temperature did not get the better outcome (Table 1, entries 10–11). When the reaction was conducted in the presence of 0.5 equiv. of TFA, the yield of product **2e** was decreased to 50% (Table 1, entry 12). Furthermore, reducing the amount of hydrazine hydrate had a negative impact on the reaction (Table 1, entry 13). Considering TFBen could generate three times as much CO per molecule, increasing the amount of TFBen to 1.0 equiv. only gave the comparable result (Table 1, entry 14). In addition, the inert atmosphere also had a negligible effect on the reaction (Table 1, entry 15).

**TABLE 1:** Optimization of reaction Conditions^a^

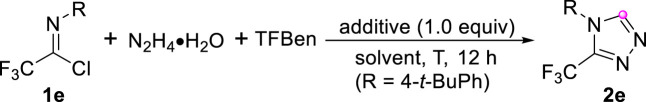				
Entry	Additive	Solvent	Temp (^o^C)	Yield (%)^b^
1	TsOH H_2_O	Toluene	100	53
2	TfOH	Toluene	100	38
3	PivOH	Toluene	100	15
**4**	**TFA**	**Toluene**	**100**	**83**
5	TFA	THF	100	52
6	TFA	DCE	100	62
7	TFA	DMSO	100	17
8	TFA	DMF	100	51
9	TFA	1,4-dioxane	100	66
10	TFA	Toluene	80	65
11	TFA	Toluene	120	77
12	TFA	Toluene	100	50^c^
13	TFA	Toluene	100	65^d^
14	TFA	Toluene	100	85^e^
15	TFA	Toluene	100	80^f^

a Reaction conditions: **1a** (0.2 mmol), N_2_H_4_•H_2_O (80%) (0.3 mmol), TFBen (0.1 mmol), additive (1.0 equiv) in solvent (2.0 ml) under air at 100°C for 12 h.

b Isolated yields.

c TFA (0.5 equiv).

d N_2_H_4_•H_2_O (80%) (0.2 mmol).

e TFBen (0.2 mmol).

f Under N_2_ atmosphere.

Having established the optimized conditions, the generality and limitation of the protocol was investigated and the result was summarized in Table 2. To our delight, the reaction exhibited good substrates compatibility, as demonstrated that diverse trifluoroacetimidoyl chlorides were smoothly tolerated in the reaction (**2a-p**). The reaction was not sensitive to the steric hindrance and the comparable reactivity was observed regarding the trifluoroacetimidoyl chlorides bearing *ortho*, *meta* or *para* substituents located at the aryl ring (**2b-d**). In general, the trifluoroacetimidoyl chlorides with electron-rich groups (**2b-g**) showed higher reactivity than that of substrates with electron-deficient groups (**2h-k**). The naphthalene ring could be successfully incorporated into the 1,2,4-triazole products (**2l** and **2m**) in 75%–78% yields. In addition, other perfluoroalkyl substituted imidoyl chlorides were also amenable to the current reaction system, providing the corresponding 1,2,4-triazoles **2n-p** with perfluoroalkyl group in acceptable yields.

**TABLE 2:** Scope of trifluoroacetimidoyl chlorides.

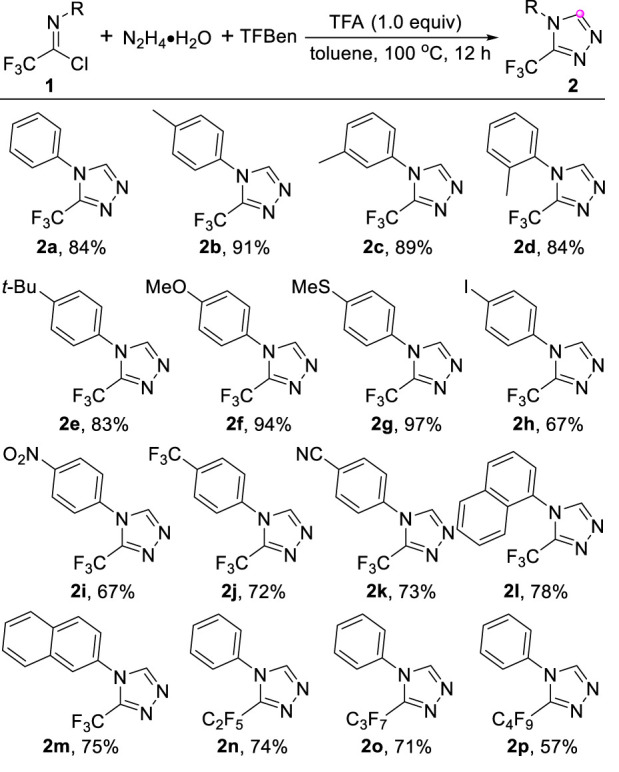

Reaction conditions: 1 (0.2 mmol), N2H4•H2O (80%) (0.3 mmol), TFBen (0.1 mmol), TFA (1.0 equiv) in toluene (2.0 ml) under air at 100 o 300°C for 12 h. Isolated yields.

**FIGURE 3:**
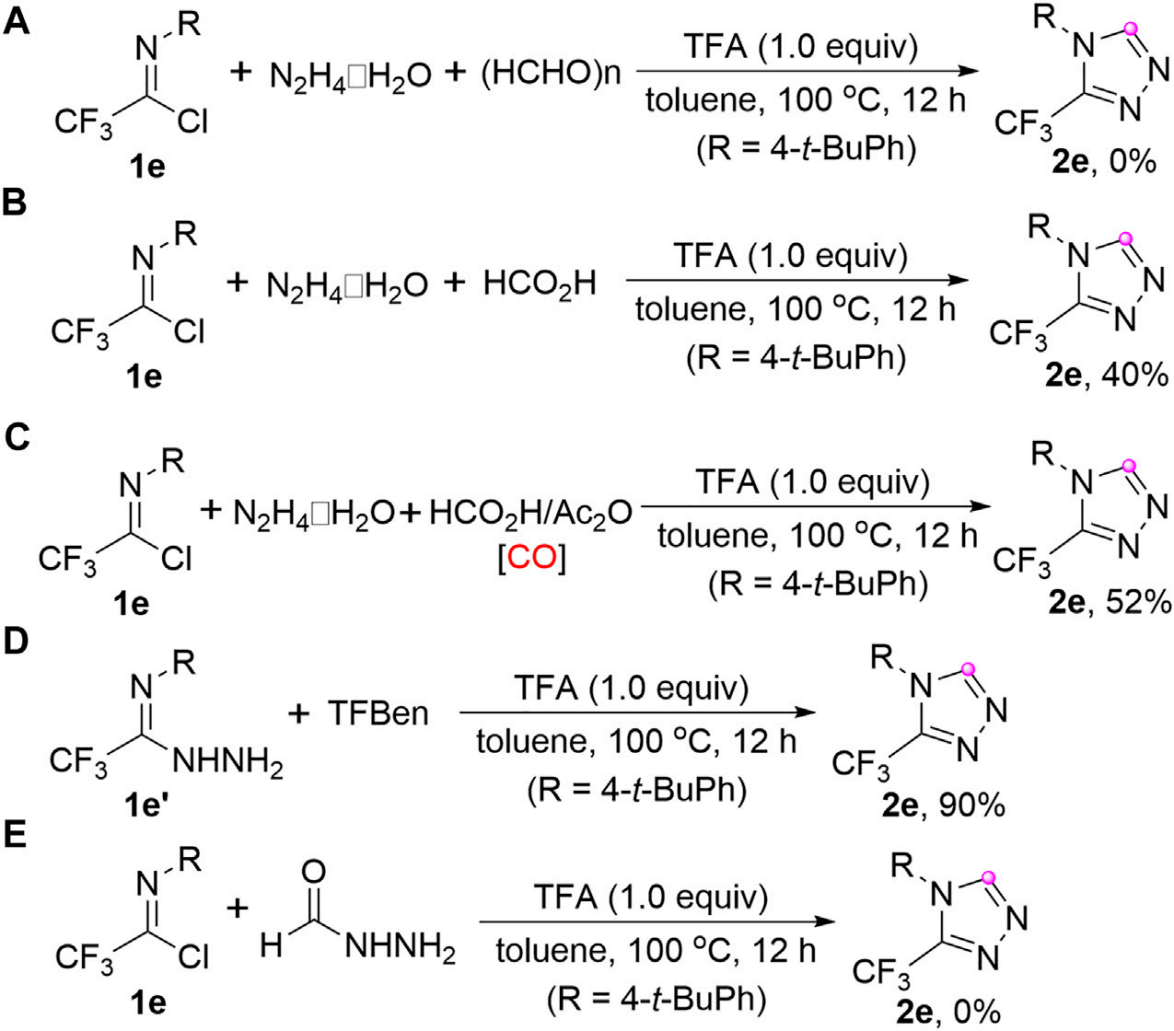
Control experiments.

Several control experiments were performed to have a deep understanding of the reaction mechanism (Figure 3). First, the replacement of TFBen with formaldehyde totally inhibited the reaction (Figure 3A), whereas the formic acid could participate in the reaction to give the product **2e** in 40% yield (Figure 3B). Then, another commonly used CO surrogate, HCO_2_H/Ac_2_O, was applied to the reaction for producing **2e** in 52% yield (Figure 3C). The above results revealed that the active carbonyl unit was released and subsequently coupled with trifluoroacetimidoyl chloride and hydrazine hydrate. The reaction of trifluoroacetimidohydrazide **1e’** with TFBen could furnish the target product **2e** in high yield, suggesting the intermediacy of **1e’** (Figure 3D). When the reaction was carried out between trifluoroacetimidoyl chloride **1e** and formhydrazide under the standard conditions, no desired product **2e** was detected (Figure 3E), which showed the hydrazine hydrate might initially couple with **1e** to form trifluoroacetimidohydrazide **1e’** and formhydrazide not acted as the reaction intermediate.

**FIGURE 4:**
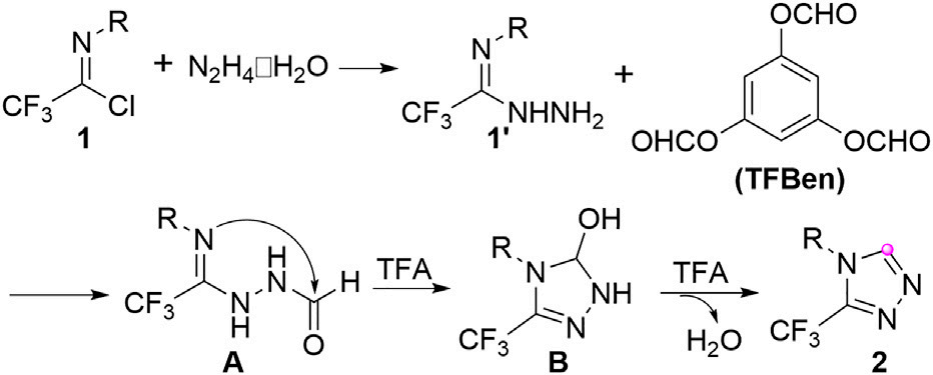
Plausible reaction mechanism.

Based on the mechanistic observations and previously reported literatures (Yin, et al., 2018; Tang, et al., 2021), a plausible reaction mechanism was proposed as outlined in Figure 4. Initially, the coupling of trifluoroacetimidoyl chloride **1** and hydrazine hydrate could readily deliver trifluoroacetimidohydrazide **1’**, which reacted with TFBen to give *N*-formyl imidohydrazide **A**. Then, the intramolecular nucleophilic addition occurred to lead to the five-membered heterocyclic intermediate **B**, followed by the dehydration process with the assistance of TFA to provide the final 3-trifluoromethyl-1,2,4-triazole products **2** and release a molecule of water.

**FIGURE 5:**
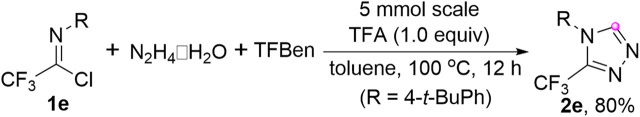
Scale up reaction.

To probe the application potential of this protocol, the reaction was performed at 5 mmol scale and the product **2e** was isolated without obvious loss of efficiency (Figure 5). Due to the excellent pharmaceutical activity of the scaffold, the present method offers the opportunity for the further study towards the toxicity risk assessment and structure-activity relationship of the pharmaceuticals containing trifluoromethyl-1,2,4-triazole cores.

## Conclusion

In conclusion, we have developed a facile and efficient strategy for the assembly of pharmaceutically valuable 3-trifluoromethyl-1,2,4-triazoles through metal-free multi-component reaction of trifluoroacetimidoyl chloride, hydrazine hydrate and TFBen. Notable features of this methodology include readily available reagents, convenient operating conditions, broad substrate scope, high efficiency, and scalability. Further studies toward the synthesis of functionalized heterocycles in a simple manner are underway.

## Data Availability

The original contributions presented in the study are included in the article/Supplementary Material, further inquiries can be directed to the corresponding authors.
