# Supplementary material for: Metal-free synthesis of 3-trifluoromethyl-1,2,4-triazoles via multi-component reaction of trifluoroacetimidoyl chlorides, hydrazine hydrate and benzene-1,3,5-triyl triformate
Source: Front Chem. 2022 Sep 20;10:1013977. doi: 10.3389/fchem.2022.1013977 (PMC9531263; doi:10.3389/fchem.2022.1013977)
Supplement: Supplementary file 1 [file DataSheet1.pdf]

Metal-Free Synthesis of 3-Trifluoromethyl-1,2,4-Triazoles via Multi-component Reaction of Trifluoroacetimidoyl Chlorides, Hydrazine Hydrate and Benzene-1,3,5-triyl Triformate

Binjie Wang,[1] Yue Sun,[2] An Cheng,[1] Yeanlun Zhu,[1] Jiye Wang[1]*, Zhengkai Chen[2]*, Xiao-Feng Wu[3,4]*

1. Key Laboratory of Drug Prevention and Control Technology of Zhejiang Province, The Department of Criminal Science and Technology, Zhejiang Police College, Hangzhou, 310053, China

2. Department of Chemistry, Key Laboratory of Surface & Interface Science of Polymer Materials of Zhejiang Province, Zhejiang Sci-Tech University, Hangzhou 310018, China

3. Dalian National Laboratory for Clean Energy, Dalian Institute of Chemical Physics, Chinese Academy of Sciences, Dalian 116023, Liaoning, China

4. Leibniz-Institut für Katalyse e. V., Albert-Einstein-Straβe 29a, 18059 Rostock, Germany

E-mail: wangjiye@zjpc.edu.cn

zkchen@zstu.edu.cn

xwu2020@dicp.ac.cn

Contents

1. General Information......S2
   1.1 Preparation of Trifluoroacetimidoyl Chlorides **1**......S2
   1.2 Preparation of Trifluoroacetimidohydrazide **1e'** S3
2. Experimental Procedures S3
   2.1 General Procedure for the Synthesis of Products **2** S3
   2.2 Control Experiments......S4
   2.3 Scale-up Reaction S6
3 Characterization Data of the Corresponding Products......S7
4 References......S15
5 Copy of ^{1}H, ^{13}C and ^{19}F NMR Spectra of Products......S16

1. General Information

Unless otherwise noted, all reactions were carried out under N_2 atmosphere. All reagents were from commercial sources and used as received without further purification. All solvents were dried by standard techniques and distilled prior to use. Column chromatography was performed on silica gel (200-300 meshes) using petroleum ether (bp. 60~90 °C) and ethyl acetate as eluent. ^{1}NMR spectra were recorded on a Bruker Avance operating at for ^{1}H NMR at 400 MHz, ^{13}C NMR at 100 MHz and spectral data were reported in ppm relative to tetramethylsilane (TMS) as internal standard and $CDCl_3$ (^{1}H NMR δ 7.26, ^{13}C NMR δ 77.16) or DMSO-D_6 (^{1}H NMR δ 2.50, ^{13}C NMR δ 39.52) as solvent. All coupling constants (*J*) are reported in Hz. The following abbreviations were used to describe peak splitting patterns when appropriate: s = singlet, d = doublet, dd = double doublet, ddd = double doublet of doublets, t = triplet, dt = double triplet, q = quatriplet, m = multiplet, br = broad. Mass spectroscopy data of the products were collected on an HRMS-TOF instrument or Waters TOFMS GCT Premier using EI or ESI ionization. Melting points were measured with WRR digital point apparatus and not corrected.

1.1 Preparation of Trifluoroacetimidoyl Chlorides[1]

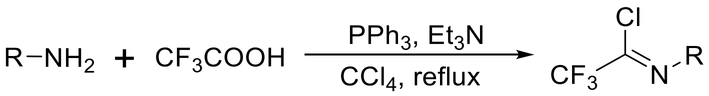

A 100 mL two-necked flask equipped with a septum cap, a condenser, and a Teflon-coated magnetic stir bar was charged with PPh_3 (9.84 g, 37.5 mmol), Et_3N (2.1 mL, 15 mmol), CCl_4 (20.0 mL), and TFA (1.2 mL, 15 mmol). After the solution was stirred for about 10 min (ice bath), amine (15 mmol) dissolved in CCl_4 (20.0 mL) was added. The mixture was then refluxed under stirring (3 h). After the reaction was completed, residual solid Ph_3PO, PPh_3 and Et_3N-HCl were washed with petroleum ether several times. Then the petroleum ether was filtered and concentrated under vacuum. The crude product was purified by column chromatography on silica gel or neutral alumina to afford the corresponding trifluoroacetimidoyl chloride products.

1.2 Preparation of Trifluoroacetimidohydrazide 1e'

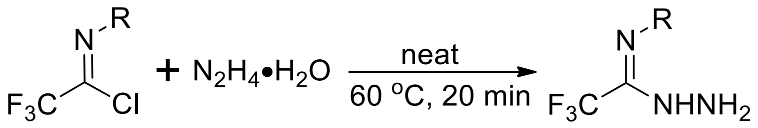

A 15 mL *In-Ex* tube equipped with a diaphragm cover, a condenser and a Teflon-coated magnetic stir bar was charged with trifluoroacetimidoyl chloride **1e** (3.0 mmol) and hydrazine hydrate (80%) (0.375 g, 6.0 mmol). The solution was stirred at 60 °C for about 20 minutes. The crude product is then purified directly by column chromatography on silica gel to obtain the corresponding trifluoroacetimidohydrazide **1e'** in almost quantitative yield.

2. Experimental Procedures

2.1 General Procedure for the Synthesis of Products 2

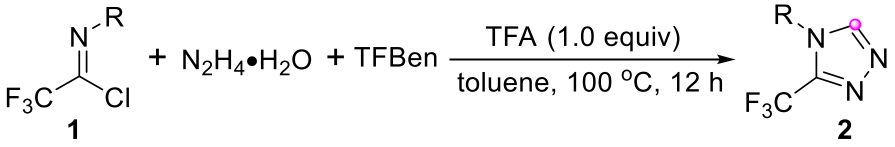

Under air atmosphere, trifluoroacetimidoyl chloride **1** (0.2 mmol, 1.0 equiv), hydrazine hydrate (80%) (0.3 mmol, 1.5 equiv), TFBen (0.1 mmol, 0.5 equiv), TFA (22.8 mg, 0.2 mmol, 1.0 equiv) and toluene (2.0 mL) were added to an oven-dried 15 mL *In-Ex* tube. Then the tube was sealed and the mixture was stirred at 100 °C (oil bath) for 12 h. After the reaction was completed, the mixture was slowly cooled to room temperature, and extracted with EtOAc for three times (3×10 mL). The extract was combined and concentrated under vacuum. The residue was purified by column chromatography on silica gel (petroleum ether/EtOAc) to yield the 3-trifluoromethyl-1,2,4-triazole products **2**.

2.2 Control Experiments

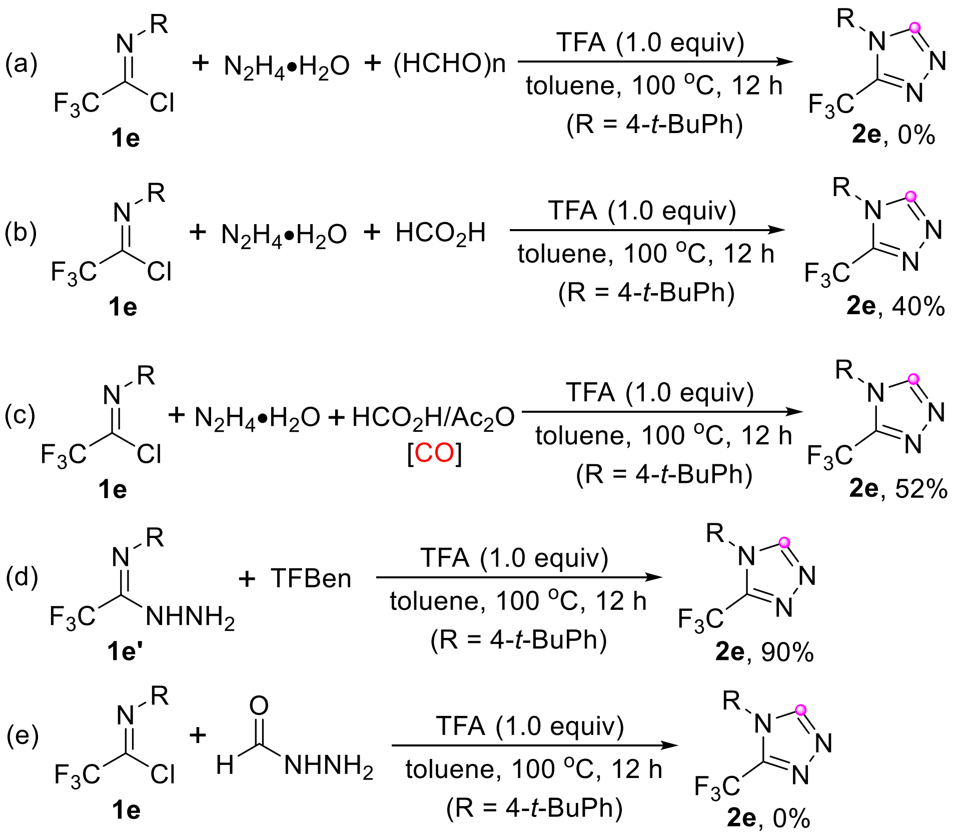

Eq. a: Under air atmosphere, trifluoroacetimidoyl chloride **1e** (52.6 mg, 0.2 mmol, 1.0 equiv), hydrazine hydrate (80%) (18.7 mg, 0.3 mmol, 1.5 equiv), (HCHO)n (12 mg, 0.4 mmol, 2.0 equiv), TFA (22.8 mg, 0.2 mmol, 1.0 equiv) and toluene (2.0 mL) were added to an oven-dried 15 mL *In-Ex* tube. Then the tube was sealed and the mixture was stirred at 100 °C (oil bath) for 12 h. After the reaction was completed, the mixture was slowly cooled to room temperature, and extracted with EtOAc for three times (3 × 10 mL). The extract was combined and concentrated under vacuum. The desired product **2e** was not observed.

Eq. b: Under air atmosphere, trifluoroacetimidoyl chloride **1e** (52.6 mg, 0.2 mmol, 1.0 equiv), hydrazine hydrate (80%) (18.7 mg, 0.3 mmol, 1.5 equiv), HCOOH (18.4 mg, 0.4 mmol, 2.0 equiv), TFA (22.8 mg, 0.2 mmol, 1.0 equiv) and toluene (2.0 mL) were added to an oven-dried 15 mL *In-Ex* tube. Then the tube was sealed and the mixture was stirred at 100 °C (oil bath) for 12 h. After the reaction was completed, the mixture was slowly cooled to room temperature, and extracted with EtOAc for three times (3 × 10 mL). The extract was combined and concentrated under vacuum. The residue was purified by column

chromatography on silica gel (petroleum ether/EtOAc) to yield the 3-trifluoromethyl-1,2,4-triazole product **2e** as a white solid in 40% yield.

Eq. c: Under air atmosphere, trifluoroacetimidoyl chloride **1e** (52.6 mg, 0.2 mmol, 1.0 equiv), hydrazine hydrate (80%) (18.7 mg, 0.3 mmol, 1.5 equiv), HCOOH/Ac_2O (0.15 mL, 1.0 mmol, 5.0 equiv, HCOOH and Ac_2O stirred for 10 minutes), TFA (22.8 mg, 0.2 mmol, 1.0 equiv) and toluene (2.0 mL) were added to an oven-dried 15 mL *In-Ex* tube. Then the tube was sealed and the mixture was stirred at 100 °C (oil bath) for 12 h. After the reaction was completed, the mixture was slowly cooled to room temperature, and extracted with EtOAc for three times (3×10 mL). The extract was combined and concentrated under vacuum. The residue was purified by column chromatography on silica gel (petroleum ether/EtOAc) to yield the 3-trifluoromethyl-1,2,4-triazole product **2e** as a white solid in 52% yield.

Eq. d: Under air atmosphere, trifluoroacetimidohydrazide **1e'** (51.8 mg, 0.2 mmol, 1.0 equiv), TFBen (21 mg, 0.1 mmol, 0.5 equiv), TFA (22.8 mg, 0.2 mmol, 1.0 equiv) and toluene (2.0 mL) were added to an oven-dried 15 mL *In-Ex* tube. Then the tube was sealed and the mixture was stirred at 100 °C (oil bath) for 12 h. After the reaction was completed, the mixture was slowly cooled to room temperature, and extracted with EtOAc for three times (3×10 mL). The extract was combined and concentrated under vacuum. The residue was purified by column chromatography on silica gel (petroleum ether/EtOAc) to yield the 3-trifluoromethyl-1,2,4-triazole products **2e** as a white solid in 90% yield.

Eq. e: Under air atmosphere, trifluoroacetimidoyl chloride **1e** (52.6 mg, 0.2 mmol, 1.0 equiv), $HCONHNH_2$ (12 mg, 0.2 mmol, 1.0 equiv), TFA (22.8 mg, 0.2 mmol, 1.0 equiv) and toluene (2.0 mL) were added to an oven-dried 15 mL *In-Ex* tube. Then the tube was sealed and the mixture was stirred at 100 °C (oil bath) for 12 h. After the reaction was completed, the mixture was slowly cooled to room temperature, and extracted with EtOAc for three times (3×10 mL). The extract was combined and concentrated under vacuum. The desired product **2e** was not observed.

2.3 Scale-up Reaction

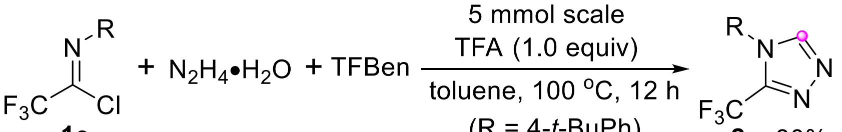

Under air atmosphere, trifluoroacetimidoyl chloride **1e** (1.315 g, 5 mmol, 1.0 equiv), hydrazine hydrate (80%) (0.469 g, 7.5 mmol, 1.5 equiv), TFBen (0.525 g, 2.5 mmol, 0.5 equiv), TFA (570 mg, 5 mmol, 1.0 equiv) and toluene (30 mL) were added to an oven-dried 100 mL *In-Ex* tube. Then the tube was sealed and the mixture was stirred at 100 °C (oil bath) for 12 h. After the reaction was completed, the mixture was slowly cooled to room temperature, and extracted with EtOAc for three times (3×50 mL). The extract was combined and concentrated under vacuum. The residue was purified by column chromatography on silica gel (petroleum ether/EtOAc) to yield the 3-trifluoromethyl-1,2,4-triazole product **2e** as a white solid in 80% yield (1.076 g).

3 Characterization Data of the Corresponding Products

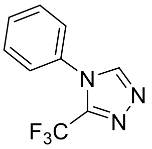

4-phenyl-3-(trifluoromethyl)-4H-1,2,4-triazole (**2a**)[2]

Upon completion the mixture was concentrated and purified via flash column chromatography (petroleum ether / ethyl acetate = 5:1, Rf = 0.2) to give the titled product **2a** as a yellow solid (35.8 mg, 84%).

^{1}H NMR (400 MHz, $CDCl_3$) δ 8.36 (s, 1H), 7.62 – 7.52 (m, 3H), 7.37 (d, J = 6.8 Hz, 2H).

^{13}C NMR (101 MHz, $CDCl_3$) δ 146.3, 144.5 (C-F, q, $^2J_{(C-F)}$ = 38.8 Hz), 132.4, 130.8, 130.0, 125.9, 118.3 (C-F, q, $^1J_{(C-F)}$ = 270.9 Hz).

^{19}F NMR (377 MHz, $CDCl_3$) δ -60.5.

M.p. 67.5 - 68.6 ^{o}C

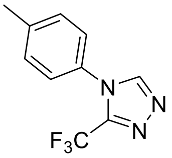

4-(p-tolyl)-3-(trifluoromethyl)-4H-1,2,4-triazole (**2b**)[2]

Upon completion the mixture was concentrated and purified via flash column chromatography (petroleum ether / ethyl acetate = 5:1, Rf = 0.3) to give the titled product **2b** as a yellow oily liquid (41.3 mg, 91%).

^{1}H NMR (400 MHz, $CDCl_3$) δ 8.33 (s, 1H), 7.34 (d, J = 8.1 Hz, 2H), 7.23 (d, J = 8.3 Hz, 2H), 2.45 (s, 3H).

^{13}C NMR (101 MHz, $CDCl_3$) δ 146.5, 144.6 (C-F, q, $^2J_{(C-F)}$ = 39.9 Hz), 141.3, 130.5, 129.8, 125.7, 118.4 (C-F, q, $^1J_{(C-F)}$ = 270.6 Hz), 21.3.

^{19}F NMR (377 MHz, $CDCl_3$) δ -60.6.

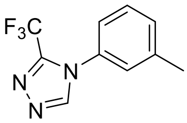

4-(m-tolyl)-3-(trifluoromethyl)-4H-1,2,4-triazole (**2c**)[2]

Upon completion the mixture was concentrated and purified via flash column chromatography (petroleum ether / ethyl acetate = 5:1, Rf = 0.3) to give the titled product **2c** as a yellow oily liquid (40.4 mg, 89%).

^{1}H NMR (400 MHz, CDCl$_3$) δ 8.33 (s, 1H), 7.46 – 7.36 (m, 2H), 7.16 (s, 2H), 2.44 (s, 3H).

^{13}C NMR (101 MHz, CDCl$_3$) δ 146.3, 144.5 (C-F, q, $^2J_{(C-F)}$ = 39.7 Hz), 140.4, 132.3, 131.6, 129.7, 126.4, 122.9, 118.2 (C-F, q, $^1J_{(C-F)}$ = 271.2 Hz), 21.3.

^{19}F NMR (377 MHz, CDCl$_3$) δ -60.5.

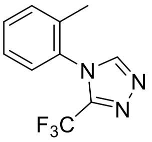

4-(o-tolyl)-3-(trifluoromethyl)-4H-1,2,4-triazole (**2d**)[2]

Upon completion the mixture was concentrated and purified via flash column chromatography (petroleum ether / ethyl acetate = 5:1, Rf = 0.3) to give the titled product **2d** as a yellow oily liquid (38.1 mg, 84%).

^{1}H NMR (400 MHz, CDCl$_3$) δ 8.27 (s, 1H), 7.49 (t, J = 7.6 Hz, 1H), 7.38 (d, J = 18.1 Hz, 2H), 7.24 (d, J = 7.8 Hz, 1H), 2.06 (s, 3H).

^{13}C NMR (101 MHz, CDCl$_3$) δ 146.2, 144.8 (C-F, q, $^2J_{(C-F)}$ = 39.4 Hz) , 135.2, 131.5, 131.3, 131.2, 127.4, 127.3, 118.1 (C-F, q, $^1J_{(C-F)}$ = 271.2 Hz), 17.0.

^{19}F NMR (377 MHz, CDCl$_3$) δ -61.8.

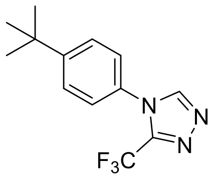

4-(4-(tert-butyl)phenyl)-3-(trifluoromethyl)-4H-1,2,4-triazole (**2e**)[2]

Upon completion the mixture was concentrated and purified via flash column chromatography (petroleum ether / ethyl acetate = 5:1, Rf = 0.3) to give the titled product **2e** as a white solid (44.7 mg, 83%).

^{1}H NMR (400 MHz, $CDCl_3$) δ 8.32 (s, 1H), 7.55 (d, *J* = 8.6 Hz, 2H), 7.28 (d, *J* = 8.5 Hz, 2H), 1.37 (s, 9H).

^{13}C NMR (101 MHz, $CDCl_3$) δ 154.3, 146.5, 144.6 (C-F, q, $^{2}J_{(C-F)}$ = 38.6 Hz), 129.7, 126.9, 125.4, 118.3 (C-F, q, $^{1}J_{(C-F)}$ = 270.9 Hz), 35.0, 31.2.

^{19}F NMR (377 MHz, $CDCl_3$) δ -60.6.

M.p. 131.5 - 132.2 ^{o}C

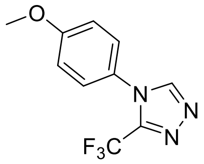

4-(4-methoxyphenyl)-3-(trifluoromethyl)-4H-1,2,4-triazole (**2f**)[2]

Upon completion the mixture was concentrated and purified via flash column chromatography (petroleum ether / ethyl acetate = 5:1, Rf = 0.3) to give the titled product **2f** as a yellow solid (45.7 mg, 94%).

^{1}H NMR (400 MHz, $CDCl_3$) δ 8.34 (s, 1H), 7.30 (d, *J* = 8.8 Hz, 2H), 7.05 (d, *J* = 8.9 Hz, 2H), 3.91 (s, 3H).

^{13}C NMR (101 MHz, $CDCl_3$) δ 161.2, 146.5, 144.8 (C-F, q, $^{2}J_{(C-F)}$ = 41.3 Hz), 127.3, 124.7, 118.3 (C-F, q, $^{1}J_{(C-F)}$ = 272.2 Hz), 115.0, 55.7.

^{19}F NMR (377 MHz, $CDCl_3$) δ -60.8.

M.p. 74.5 - 77.6 ^{o}C

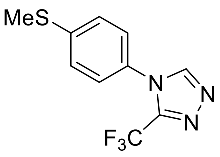

4-(4-(methylthio)phenyl)-3-(trifluoromethyl)-4H-1,2,4-triazole (**2g**)[2]

Upon completion the mixture was concentrated and purified via flash column chromatography (petroleum ether / ethyl acetate = 5:1, Rf = 0.3) to give the titled product **2g** as a white solid (50.2 mg, 97%).

^{1}H NMR (400 MHz, $CDCl_3$) δ 8.32 (s, 1H), 7.35 (d, *J* = 8.7 Hz, 2H), 7.26 (d, *J* = 8.6 Hz, 2H), 2.54 (s, 3H).

^{13}C NMR (101 MHz, $CDCl_3$) δ 146.3, 144.6 (C-F, q, $^{2}J_{(C-F)}$ = 39.4 Hz), 143.1, 128.6, 126.7, 126.2, 118.2, 15.2.

^{19}F NMR (377 MHz, $CDCl_3$) δ -60.6.

M.p. 92.4 - 95.8 °C

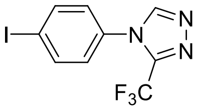

4-(4-iodophenyl)-3-(trifluoromethyl)-4H-1,2,4-triazole (**2h**)[2]

Upon completion the mixture was concentrated and purified via flash column chromatography (petroleum ether / ethyl acetate = 5:1, Rf = 0.3) to give the titled product **2h** as a yellow solid (45.4 mg, 67%).

^{1}H NMR (400 MHz, $CDCl_3$) δ 8.33 (s, 1H), 7.91 (d, *J* = 8.5 Hz, 2H), 7.12 (d, *J* = 8.5 Hz, 2H).

^{13}C NMR (101 MHz, $CDCl_3$) δ 146.0, 144.4 (C-F, q, $^{2}J_{(C-F)}$ = 40.5 Hz), 139.3, 132.0, 127.5, 118.1 (C-F, q, $^{1}J_{(C-F)}$ = 271.2 Hz), 96.8.

^{19}F NMR (377 MHz, $CDCl_3$) δ -60.4.

M.p. 151.2 - 152.5 °C

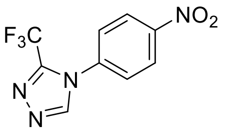

4-(4-nitrophenyl)-3-(trifluoromethyl)-4H-1,2,4-triazole **(2i)**[2]

Upon completion the mixture was concentrated and purified via flash column chromatography (petroleum ether / ethyl acetate = 5:1, Rf = 0.3) to give the titled product **2i** as a yellow solid (34.6 mg, 67%).

^{1}H NMR (400 MHz, $CDCl_3$) δ 8.47 (d, *J* = 8.9 Hz, 2H), 8.42 (s, 1H), 7.63 (d, *J* = 8.8 Hz, 2H).

^{13}C NMR (101 MHz, $CDCl_3$) δ 149.0, 145.6, 144.7 (C-F, q, $^2J_{(C-F)}$ = 37.5 Hz), 137.3, 127.2, 125.5, 118.0 (C-F, q, $^1J_{(C-F)}$ = 271.3 Hz).

^{19}F NMR (377 MHz, $CDCl_3$) δ -60.1.

M.p. 142.3 - 143.8 ^{o}C

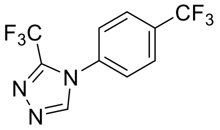

3-(trifluoromethyl)-4-(4-(trifluoromethyl)phenyl)-4H-1,2,4-triazole (**2j**)[3]

Upon completion the mixture was concentrated and purified via flash column chromatography (petroleum ether / ethyl acetate = 5:1, Rf = 0.3) to give the titled product **2j** as a white solid (40.5 mg, 72%).

^{1}H NMR (400 MHz, $CDCl_3$) δ 8.39 (s, 1H), 7.87 (d, *J* = 8.4 Hz, 2H), 7.55 (d, *J* = 8.3 Hz, 2H).

^{13}C NMR (101 MHz, $CDCl_3$) δ 145.8, 135.3, 133.4, 133.1, 127.4, 127.4, 126.6, 124.5, 121.8, 118.1 (C-F, q, $^1J_{(C-F)}$ = 271.3 Hz).

^{19}F NMR (377 MHz, $CDCl_3$) δ -60.3, -63.0.

M.p. 107.8 - 109.6 ^{o}C

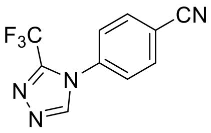

4-(3-(trifluoromethyl)-4H-1,2,4-triazol-4-yl)benzonitrile (**2k**)[3]

Upon completion the mixture was concentrated and purified via flash column chromatography (petroleum ether / ethyl acetate = 5:1, Rf = 0.3) to give the titled product **2k** as a yellow solid (34.8 mg, 73%).

^{1}H NMR (400 MHz, $CDCl_3$) δ 8.40 (s, 1H), 7.90 (d, J = 8.5 Hz, 2H), 7.57 (d, J = 8.4 Hz, 2H).

^{13}C NMR (101 MHz, $CDCl_3$) δ 145.7, 144.2 (C-F, q, $^2J_{(C\text{-}F)}$ = 39.8 Hz), 135.9, 134.1, 127.0, 118.0 (C-F, q, $^1J_{(C\text{-}F)}$ = 271.3 Hz), 116.9, 115.3.

^{19}F NMR (377 MHz, $CDCl_3$) δ -60.2.

M.p. 158.7 - 160.5 ^{o}C

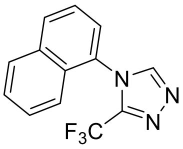

4-(naphthalen-1-yl)-3-(trifluoromethyl)-4H-1,2,4-triazole (**2l**)[2]

Upon completion the mixture was concentrated and purified via flash column chromatography (petroleum ether / ethyl acetate = 5:1, Rf = 0.3) to give the titled product **2l** as a white solid (41.0 mg, 78%).

^{1}H NMR (400 MHz, $CDCl_3$) δ 8.41 (s, 1H), 8.10 (d, J = 8.3 Hz, 1H), 8.00 (d, J = 7.9 Hz, 1H), 7.65 – 7.51 (m, 4H), 7.17 (d, J = 8.4 Hz, 1H).

^{13}C NMR (101 MHz, $CDCl_3$) δ 147.1, 145.6 (C-F, q, $^2J_{(C\text{-}F)}$ = 39.7 Hz), 134.0, 131.6, 129.5, 128.7, 128.6, 128.4, 127.6, 125.4, 125.0, 120.9, 118.2 (C-F, q, $^1J_{(C\text{-}F)}$ = 271.5 Hz).

^{19}F NMR (377 MHz, $CDCl_3$) δ -61.5.

M.p. 131.5 - 133.4 ^{o}C

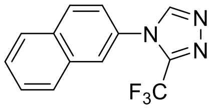

4-(naphthalen-2-yl)-3-(trifluoromethyl)-4H-1,2,4-triazole (**2m**)[2]

Upon completion the mixture was concentrated and purified via flash column chromatography (petroleum ether / ethyl acetate = 5:1, Rf = 0.4) to give the titled product **2m** as a white solid (39.5 mg, 75%).

^{1}H NMR (400 MHz, $CDCl_3$) δ 8.44 (s, 1H), 8.03 (d, *J* = 8.7 Hz, 1H), 8.00 – 7.95 (m, 1H), 7.94 – 7.90 (m, 1H), 7.88 (s, 1H), 7.69 – 7.61 (m, 2H), 7.41 (d, *J* = 8.7 Hz, 1H).

^{13}C NMR (101 MHz, $CDCl_3$) δ 146.5, 144.8 (C-F, q, $^{2}J_{(C\text{-}F)}$ = 39.4 Hz), 133.6, 132.8, 130.3, 129.6, 128.3, 128.3, 128.1, 128.0, 125.2, 122.8, 118.3 (C-F, q, $^{1}J_{(C\text{-}F)}$ = 271.2 Hz).

^{19}F NMR (377 MHz, $CDCl_3$) δ -60.5.

M.p. 118.8 - 120.5 ^{o}C

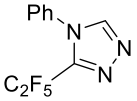

3-(perfluoroethyl)-4-phenyl-4H-1,2,4-triazole (**2n**)[2]

Upon completion the mixture was concentrated and purified via flash column chromatography (petroleum ether / ethyl acetate = 5:1, Rf = 0.4) to give the titled product **2n** as a white solid (38.9 mg, 74%).

^{1}H NMR (400 MHz, $CDCl_3$) δ 8.36 (s, 1H), 7.61 – 7.50 (m, 3H), 7.37 (d, *J* = 7.4 Hz, 2H).

^{13}C NMR (101 MHz, $CDCl_3$) δ 146.75, 143.6 (C-F, t, $^{3}J_{(C\text{-}F)}$ = 28.1 Hz), 132.6, 130.8, 129.85, 1263, 118.0 (C-F, qt, $^{2}J_{(C\text{-}F)}$ = 286.4, 34.9 Hz), 107.7 (C-F, tq, $^{1}J_{(C\text{-}F)}$ = 254.5, 40.7 Hz).

^{19}F NMR (377 MHz, $CDCl_3$) δ -108.6, -82.4.

M.p. 100.5- 102.8 ^{o}C

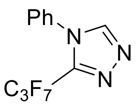

3-(perfluoropropyl)-1-phenyl-1H-1,2,4-triazole (**2o**)[2]

Upon completion the mixture was concentrated and purified via flash column chromatography (petroleum ether / ethyl acetate = 5:1, Rf = 0.3) to give the titled product **2o** as a white solid (44.5 mg, 71%).

^{1}H NMR (400 MHz, CDCl$_3$) δ 8.35 (s, 1H), 7.61 – 7.51 (m, 3H), 7.35 (d, J = 7.5 Hz, 2H).

^{13}C NMR (101 MHz, CDCl$_3$) δ 147.0, 143.7 (C-F, t, $^{4}{}_{(C\text{-}F)}$ = 27.4 Hz), 132.7, 130.8, 129.7, 126.4, 117.5 (C-F, qt, $^{3}J_{(C\text{-}F)}$ = 288.1, 33.5 Hz), 109.8 (C-F, tt, $^{2}J_{(C\text{-}F)}$ = 264.0, 34.6 Hz).

^{19}F NMR (377 MHz, CDCl$_3$) δ -125.0, -106.9, -80.0.

M.p. 86.1 - 87.7 ^{o}C

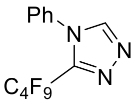

3-(perfluorobutyl)-1-phenyl-1H-1,2,4-triazole (**2p**)[2]

Upon completion the mixture was concentrated and purified via flash column chromatography (petroleum ether / ethyl acetate = 5:1, Rf = 0.4) to give the titled product **2p** as a white solid (41.4 mg, 57%).

^{1}H NMR (400 MHz, CDCl$_3$) δ 8.35 (s, 1H), 7.60 – 7.51 (m, 3H), 7.34 (d, J = 7.5 Hz, 2H).

^{13}C{^{1}H} NMR (101 MHz, CDCl$_3$) δ 147.2, 143.8 (C-F, t, $^{3}J_{(C\text{-}F)}$ = 25.0 Hz), 132.7, 130.8, 129.7, 126.5, 117.2 (C-F, tq, $^{2}J_{(C\text{-}F)}$ = 288.2, 33.1 Hz), 112.9 (C-F, tt, $^{1}J_{(C\text{-}F)}$ = 258.6, 33.5 Hz).

^{19}F NMR (377 MHz, CDCl$_3$) δ -125.5, -121.4, -106.3, -81.0.

M.p. 77.4 - 79.4 ^{o}C

4 References

(1) K. Tamura, H. Mizukami, K. Maeda, H. Watanabe and K. Uneyama, One-pot synthesis of trifluoroacetimidoyl halides, *J. Org. Chem.*, 1993, **58**, 32.

(2) H. Yang, S.-N. Lu, Y. Song, Z. Chen and X.-F. Wu, Copper-mediated [3 + 2] cycloaddition of trifluoroacetimidoyl chlorides and *N*-isocyanoiminotriphenylphosphorane for the synthesis of 3-trifluoromethyl-1,2,4-triazoles, *Org. Chem. Front.*, 2021, **8**, 5040.

(3) S.-N. Lu, J. Zhang, J. Li, Z. Chen and X.-F. Wu, Metal-free synthesis of 3-trifluoromethyl-1,2,4-triazoles via oxidative cyclization of trifluoroacetimidohydrazides with *N*,*N*-dimethylformamide as carbon synthons, *Green Synth. Catal.*, 2022, DOI: 10.1016/j.gresc.2022.06.007.

5 Copy of ^{1}H, ^{13}C and ^{19}F NMR Spectra of Products

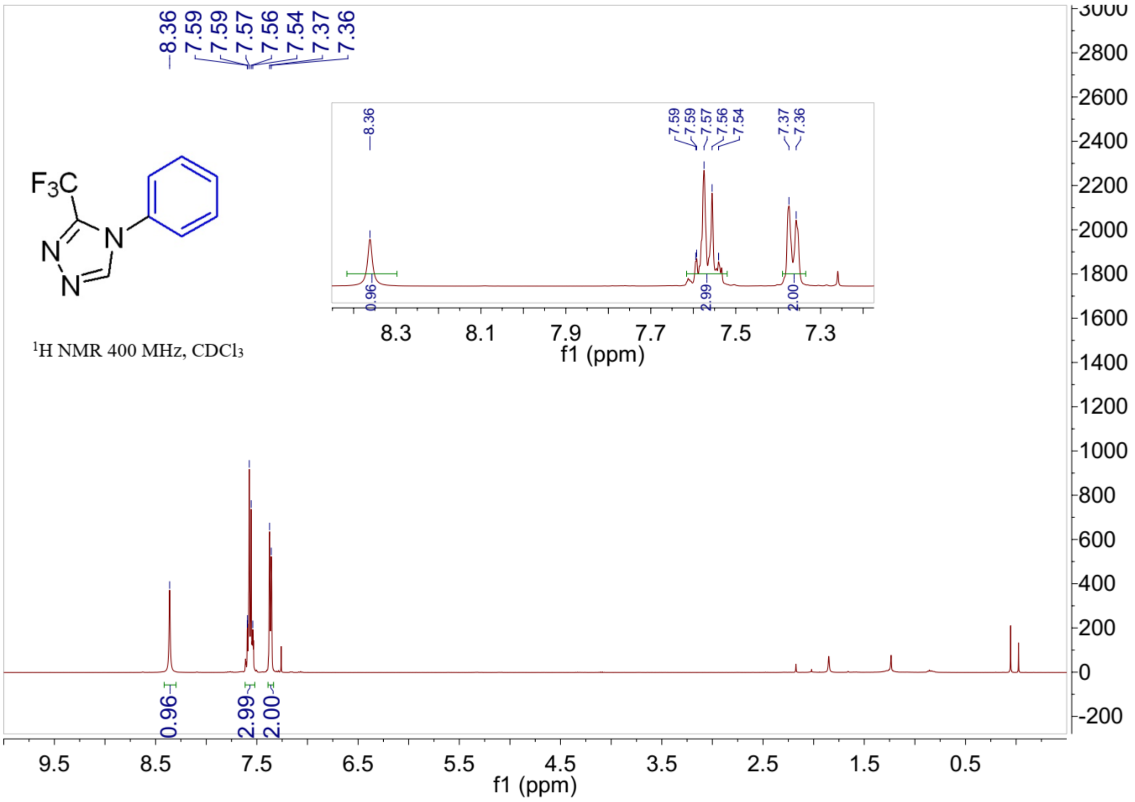

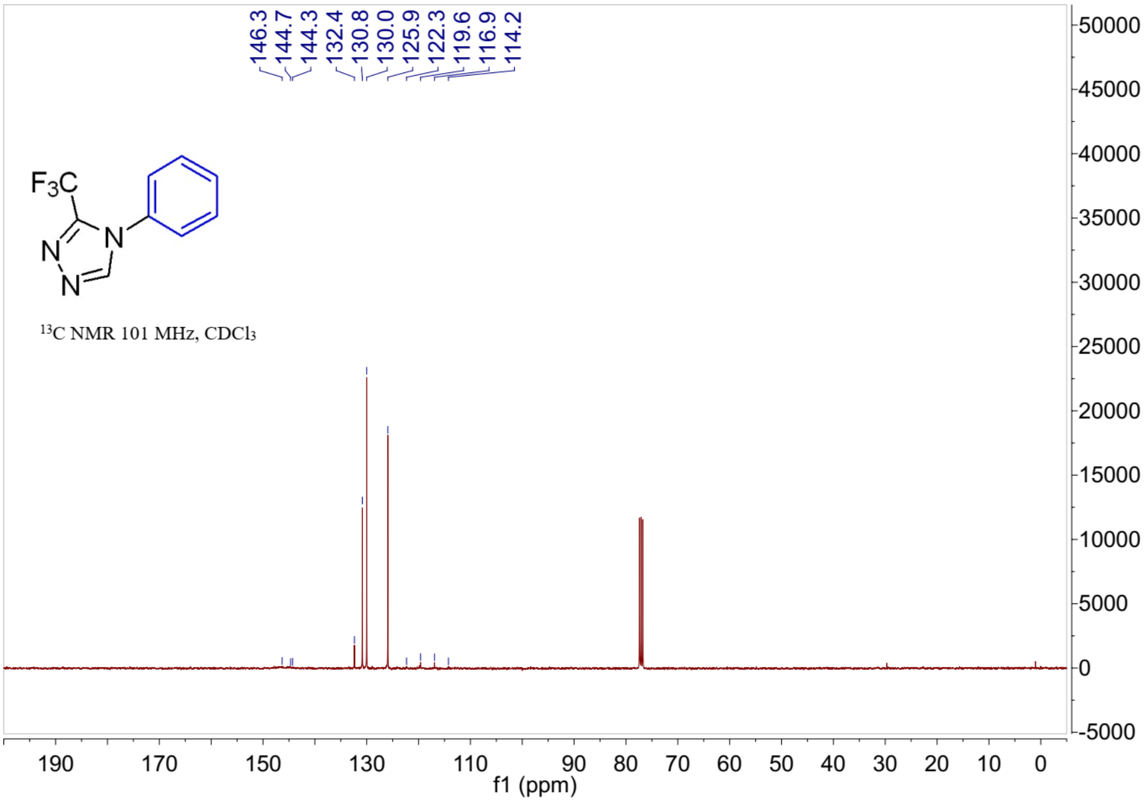

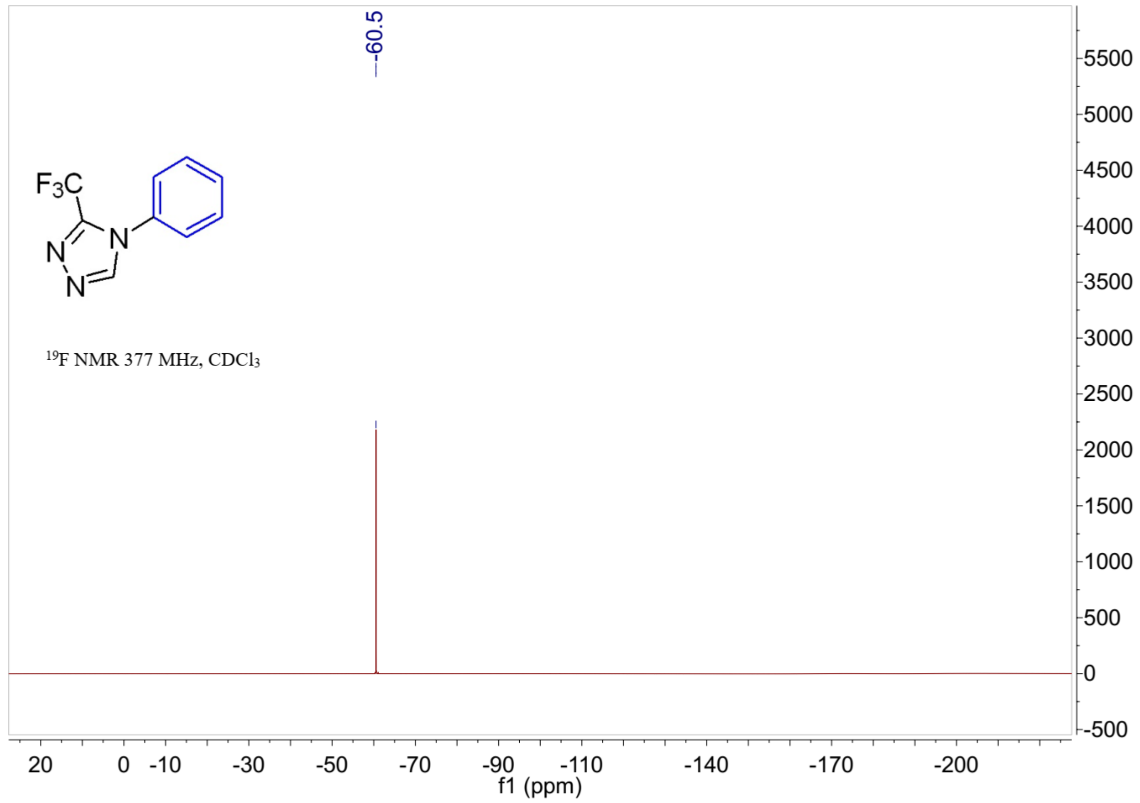

-60.5
F3C
N
N
N
19F NMR 377 MHz, CDCl3
5500
5000
4500
4000
3500
3000
2500
2000
1500
1000
500
0
-500
20
0
-10
-30
-50
-70
-90
-110
-140
-170
-200
f1 (ppm)

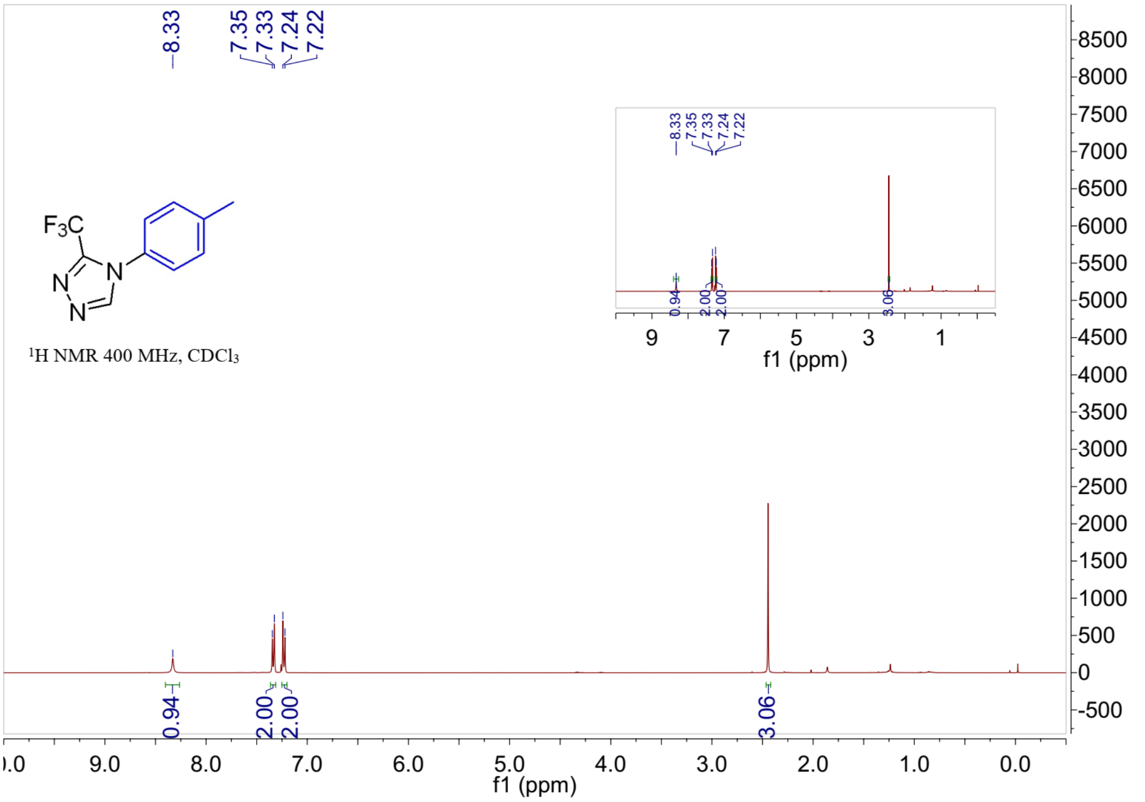
F3C
N
N
N
1H NMR 400 MHz, CDCl3
8.33
7.35
7.33
7.24
7.22
0.94
2.00
2.00
3.06
f1 (ppm)

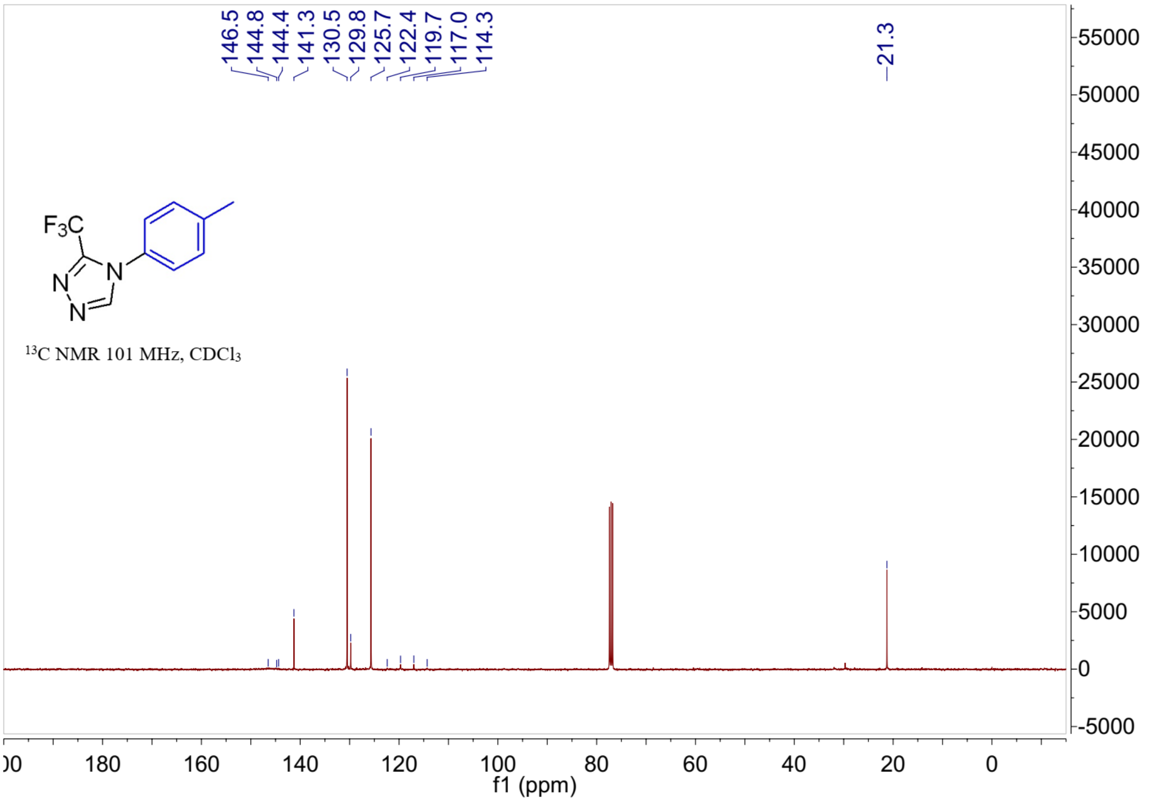
F3C
N
N
N
13C NMR 101 MHz, CDCl3
146.5
144.8
144.4
141.3
130.5
129.8
125.7
122.4
119.7
117.0
114.3
21.3
f1 (ppm)

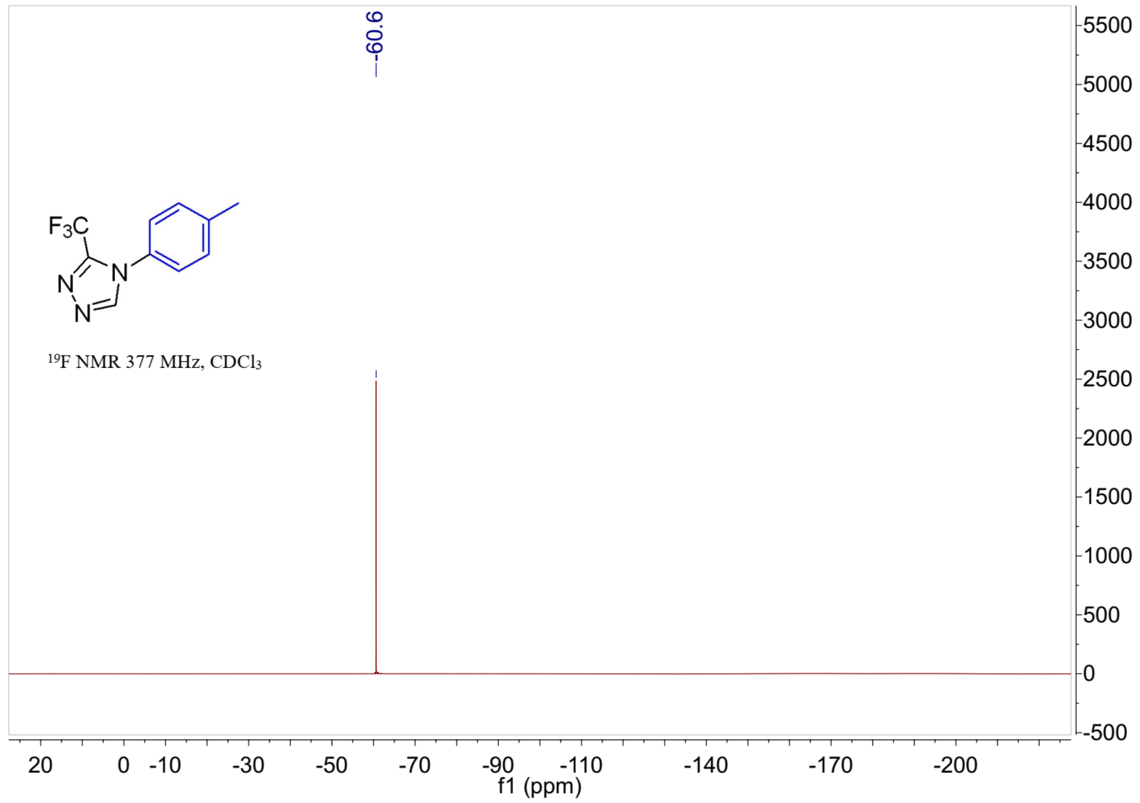
-60.6
F3C
N
N
N
19F NMR 377 MHz, CDCl3
5500
5000
4500
4000
3500
3000
2500
2000
1500
1000
500
0
-500
20
0
-10
-30
-50
-70
-90
-110
-140
-170
-200
f1 (ppm)

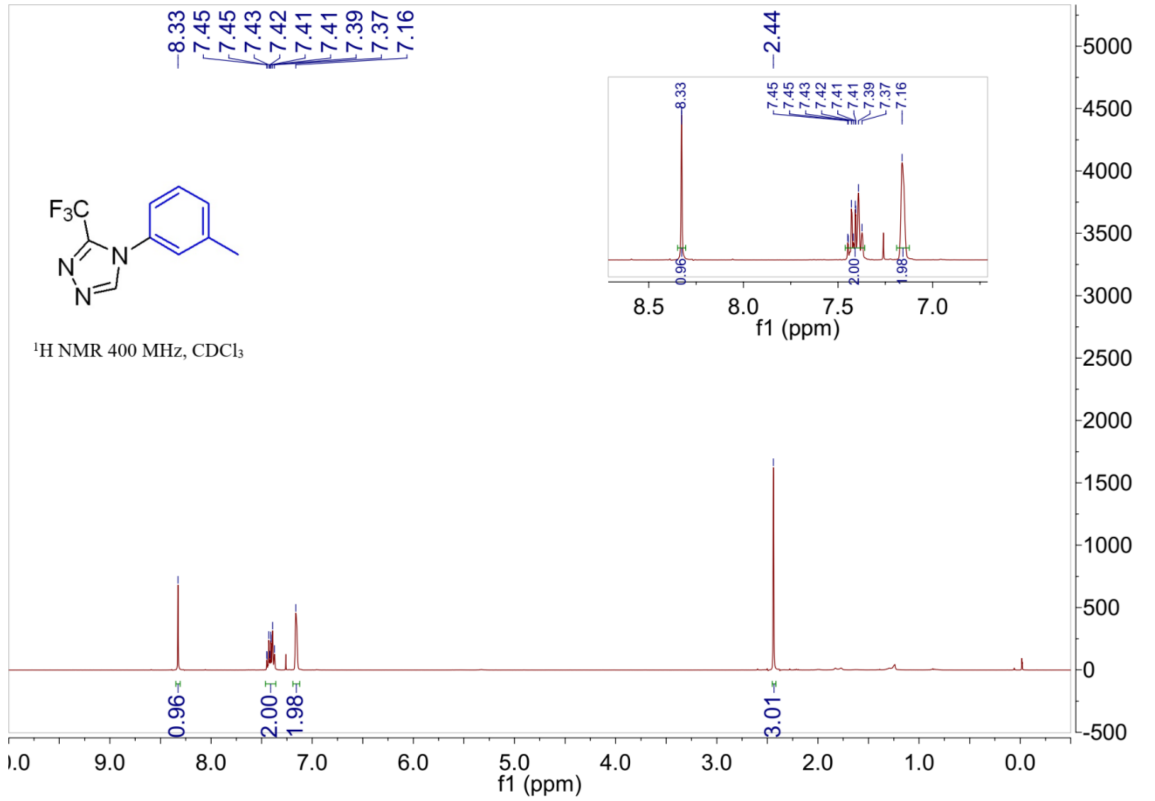
8.33
7.45
7.45
7.43
7.42
7.41
7.41
7.39
7.37
7.16
2.44
F3C
N
N
N
1H NMR 400 MHz, CDCl3
0.96
2.00
1.98
3.01
f1 (ppm)

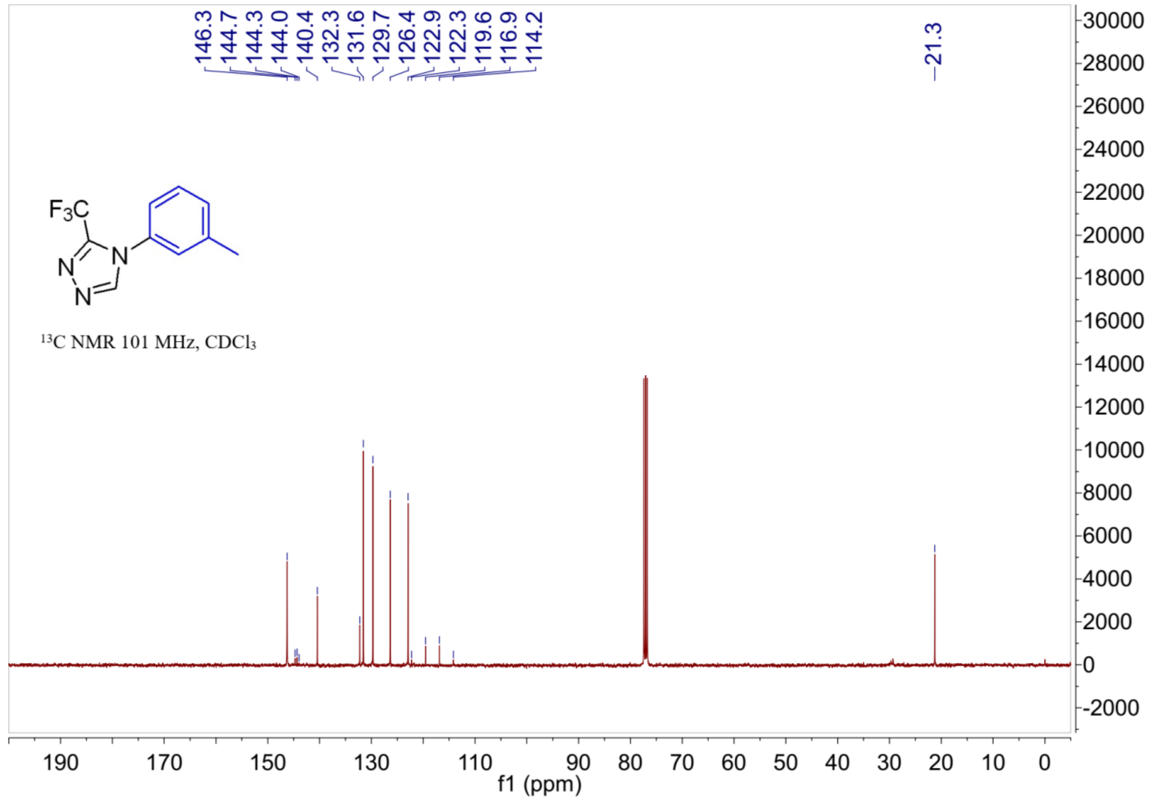
146.3
144.7
144.3
144.0
140.4
132.3
131.6
129.7
126.4
122.9
122.3
119.6
116.9
114.2
21.3
F3C
N
N
N
13C NMR 101 MHz, CDCl3
f1 (ppm)

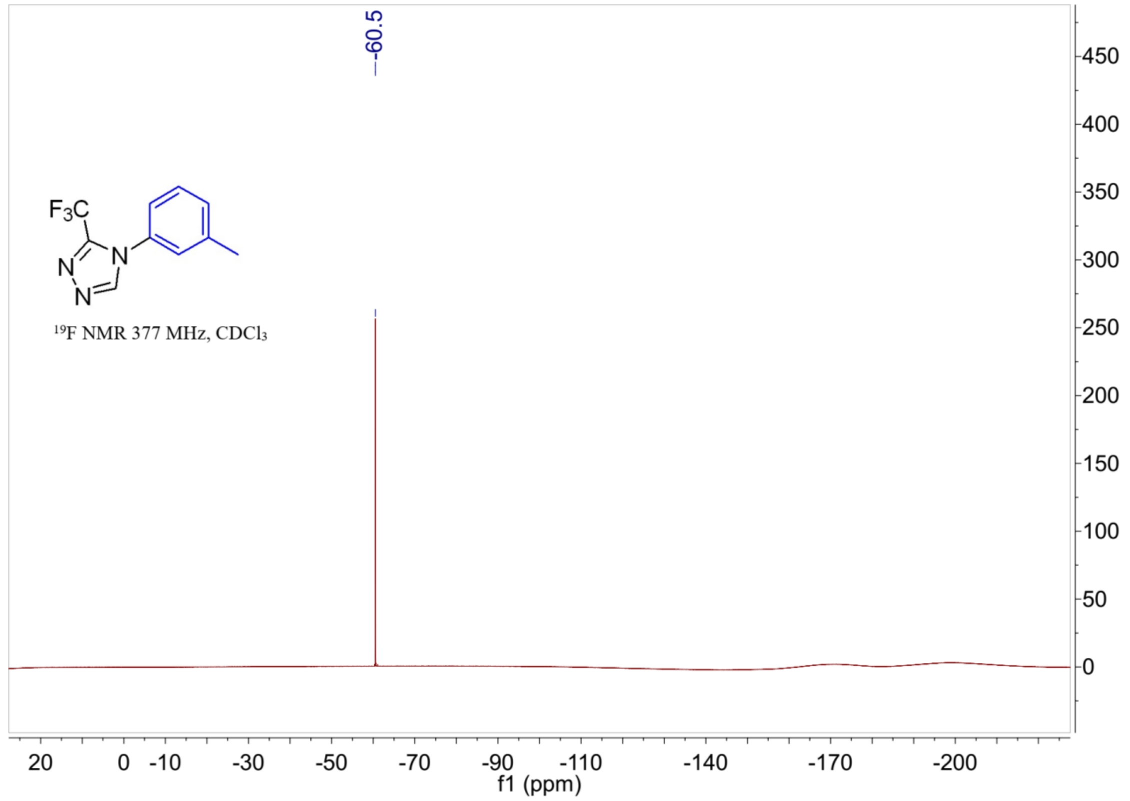
-60.5
F3C
N
N
N
19F NMR 377 MHz, CDCl3
450
400
350
300
250
200
150
100
50
0
20
0
-10
-30
-50
-70
-90
-110
-140
-170
-200
f1 (ppm)

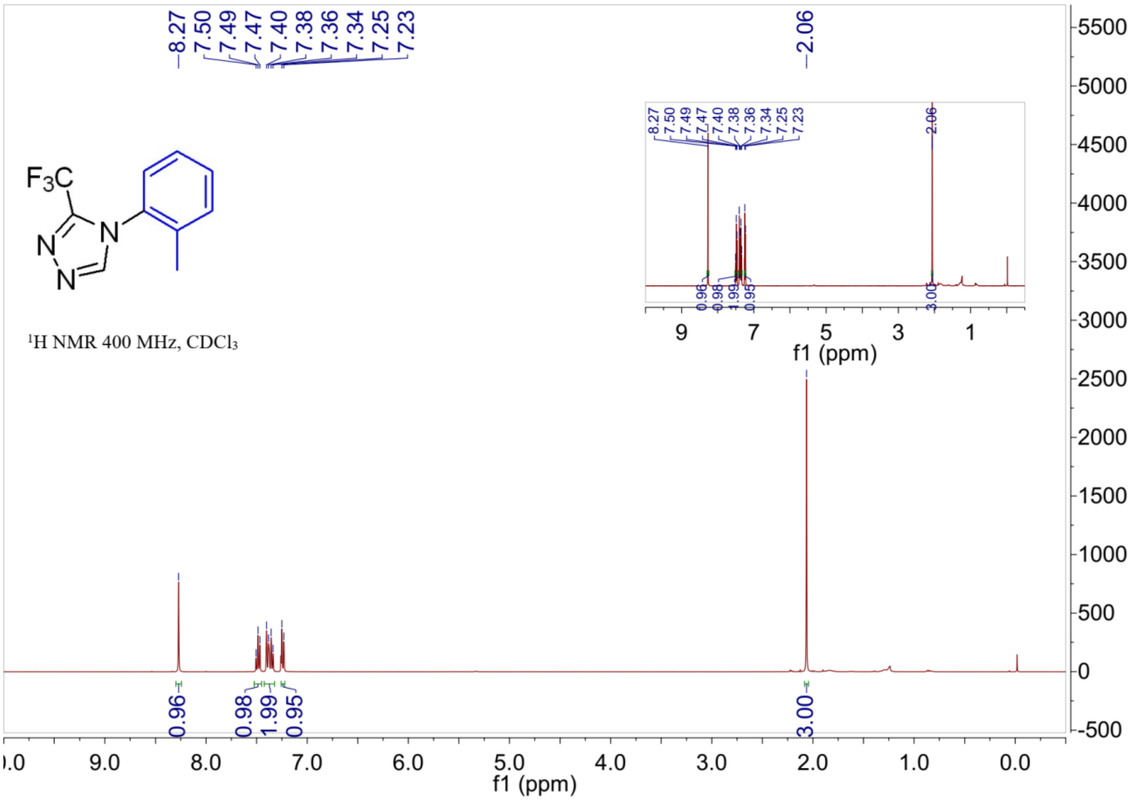
8.27 7.50 7.49 7.47 7.40 7.38 7.36 7.34 7.25 7.23
2.06
F3C
N
N
N
1H NMR 400 MHz, CDCl3
0.96 0.98 1.99 0.95
3.00
f1 (ppm)

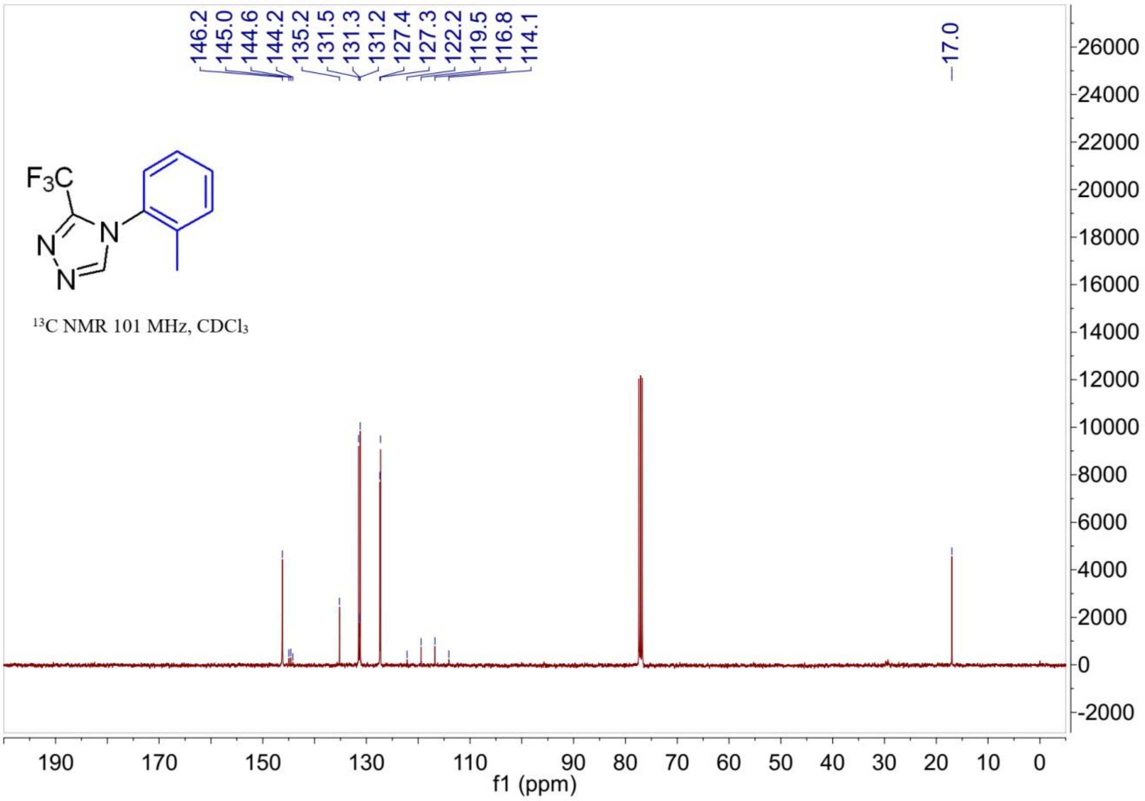
146.2 145.0 144.6 144.2 135.2 131.5 131.3 131.2 127.4 127.3 122.2 119.5 116.8 114.1
17.0
F3C
N
N
N
13C NMR 101 MHz, CDCl3
f1 (ppm)

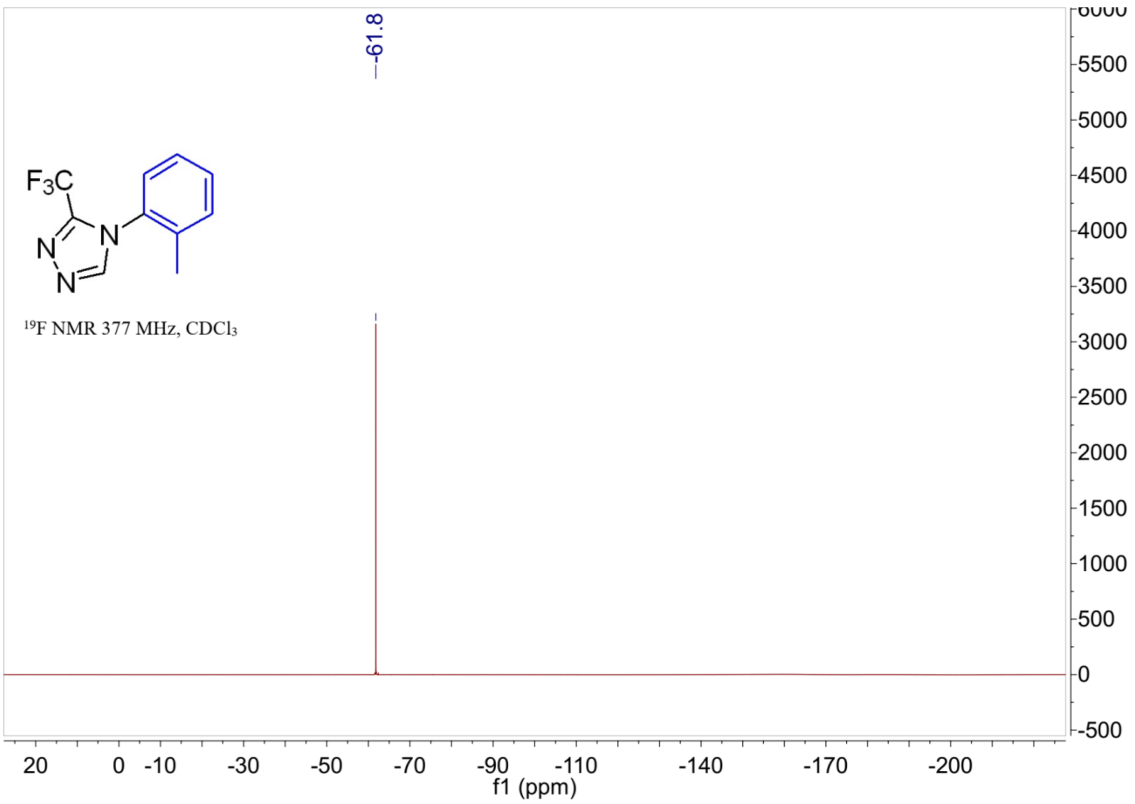

-61.8
F3C
N
N
N
19F NMR 377 MHz, CDCl3
6000
5500
5000
4500
4000
3500
3000
2500
2000
1500
1000
500
0
-500
20
0
-10
-30
-50
-70
-90
-110
-140
-170
-200
f1 (ppm)

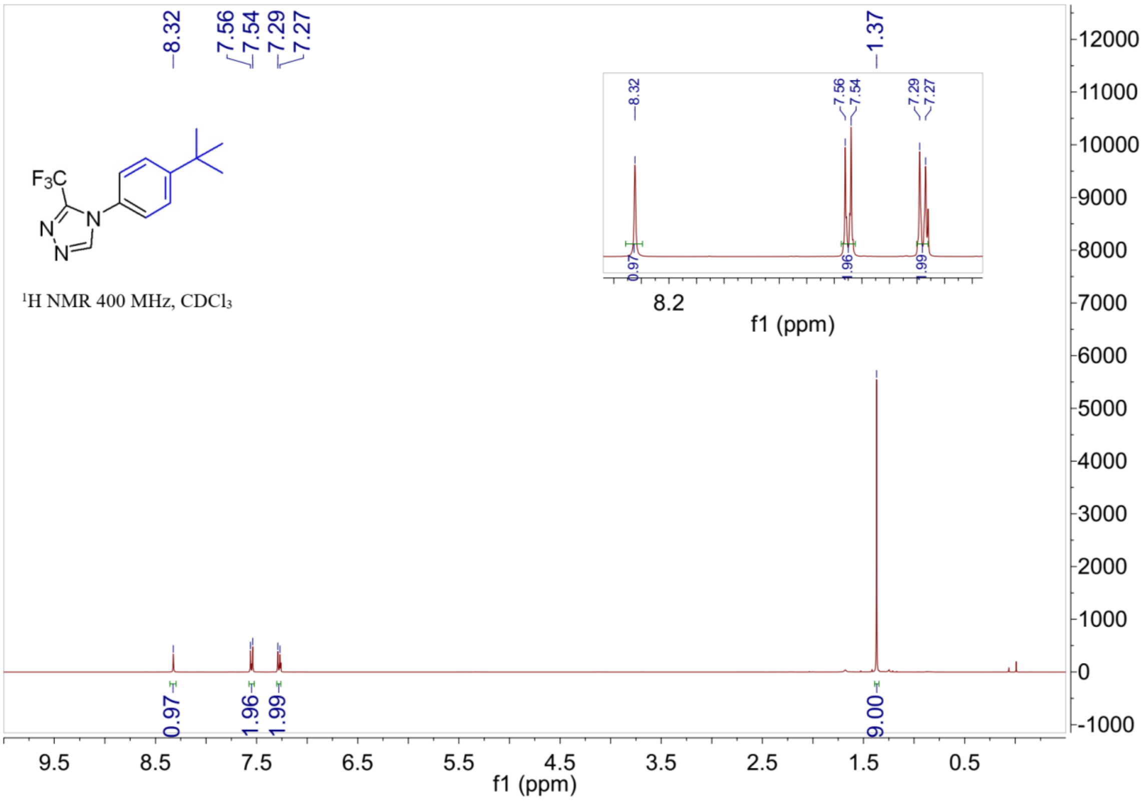
8.32
7.56
7.54
7.29
7.27
1.37
F3C
N
N
N
N
1H NMR 400 MHz, CDCl3
0.97
1.96
1.99
9.00
8.2
f1 (ppm)
f1 (ppm)

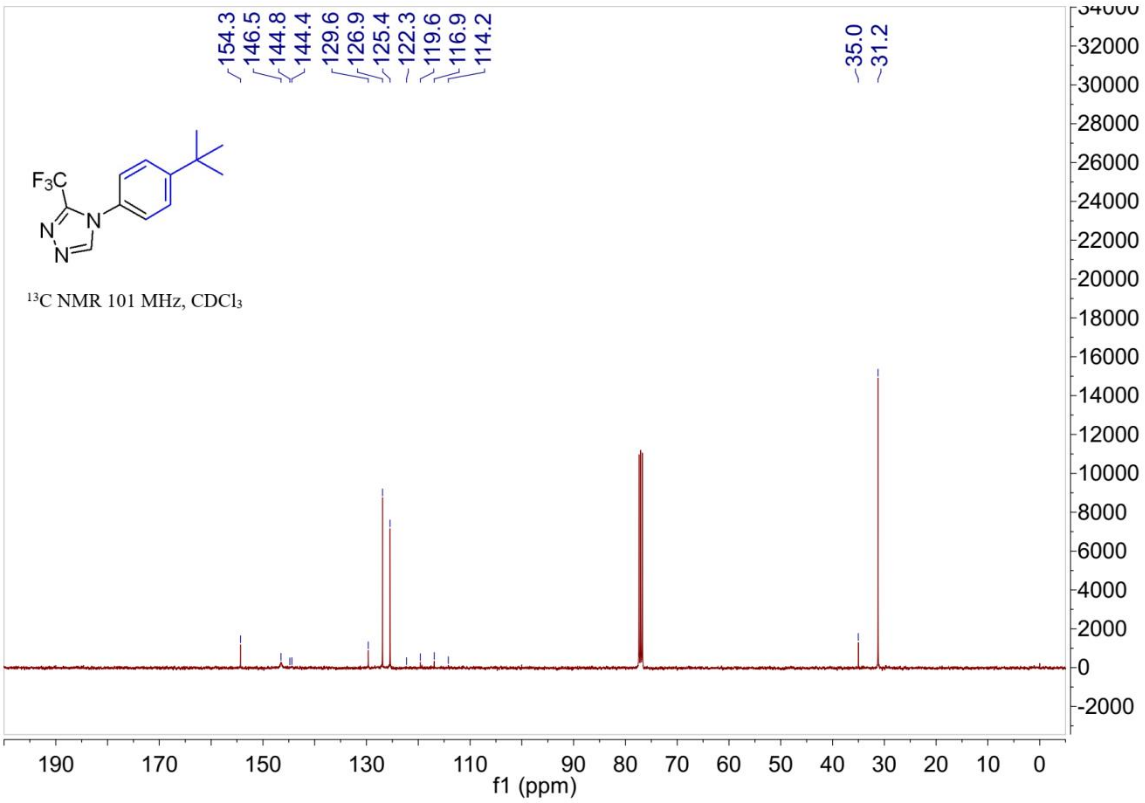
154.3
146.5
144.8
144.4
129.6
126.9
125.4
122.3
119.6
116.9
114.2
35.0
31.2
F3C
N
N
N
13C NMR 101 MHz, CDCl3
f1 (ppm)

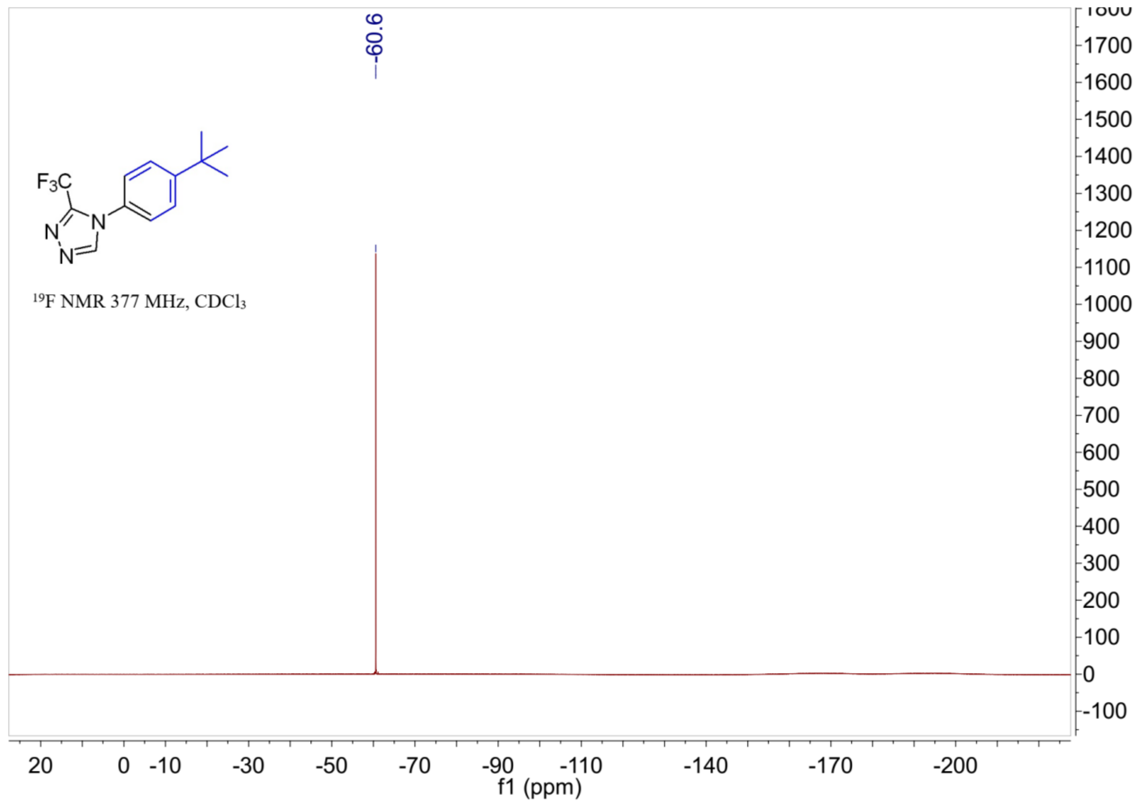
-60.6
F_3C
N
N
N
^{19}F NMR 377 MHz, $CDCl_3$
f1 (ppm)

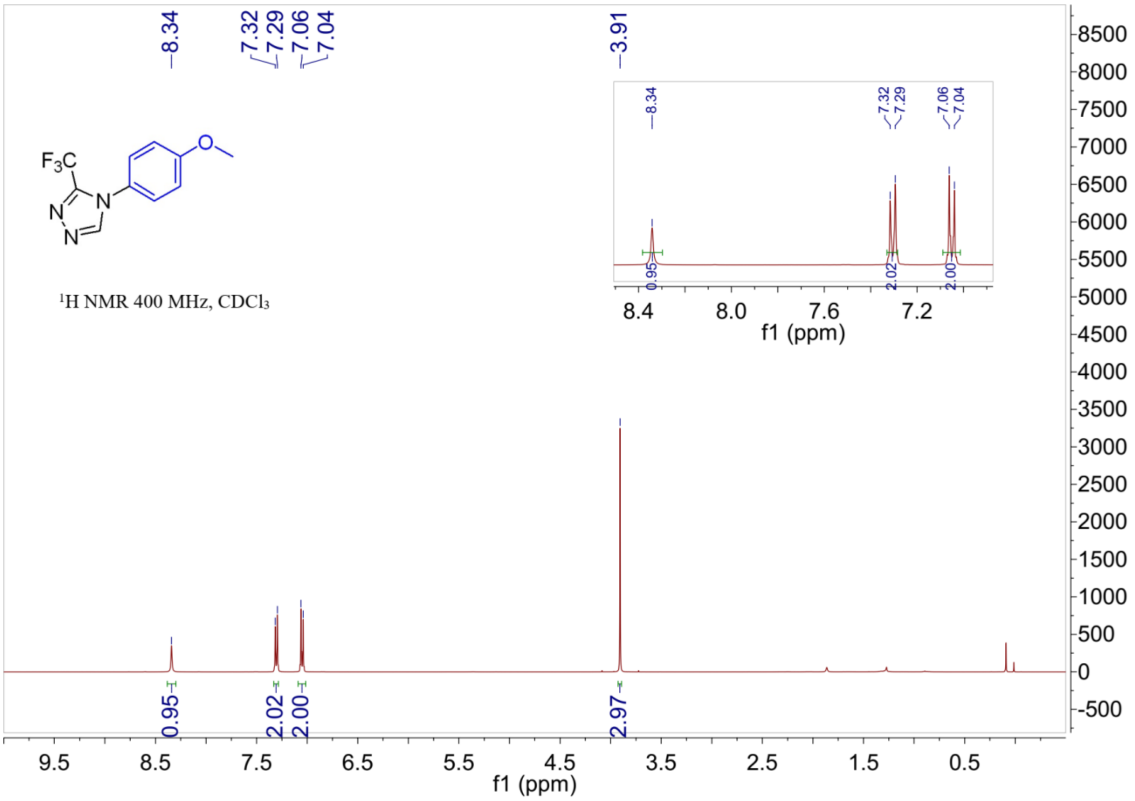

8.34
7.32
7.29
7.06
7.04
3.91
F_3C
N
N
N
O
^{1}H NMR 400 MHz, $CDCl_3$
0.95
2.02
2.00
2.97
f1 (ppm)

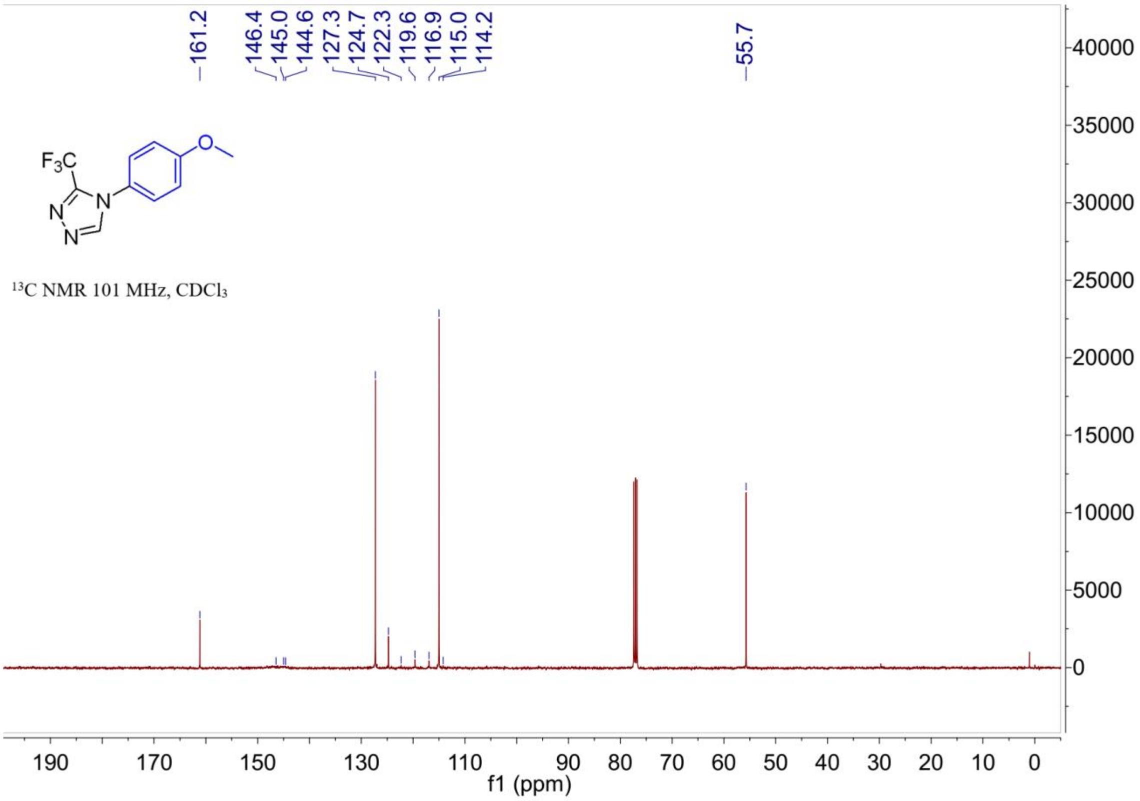

161.2
146.4
145.0
144.6
127.3
124.7
122.3
119.6
116.9
115.0
114.2
55.7
F_3C
N
N
N
O
^{13}C NMR 101 MHz, $CDCl_3$
f1 (ppm)

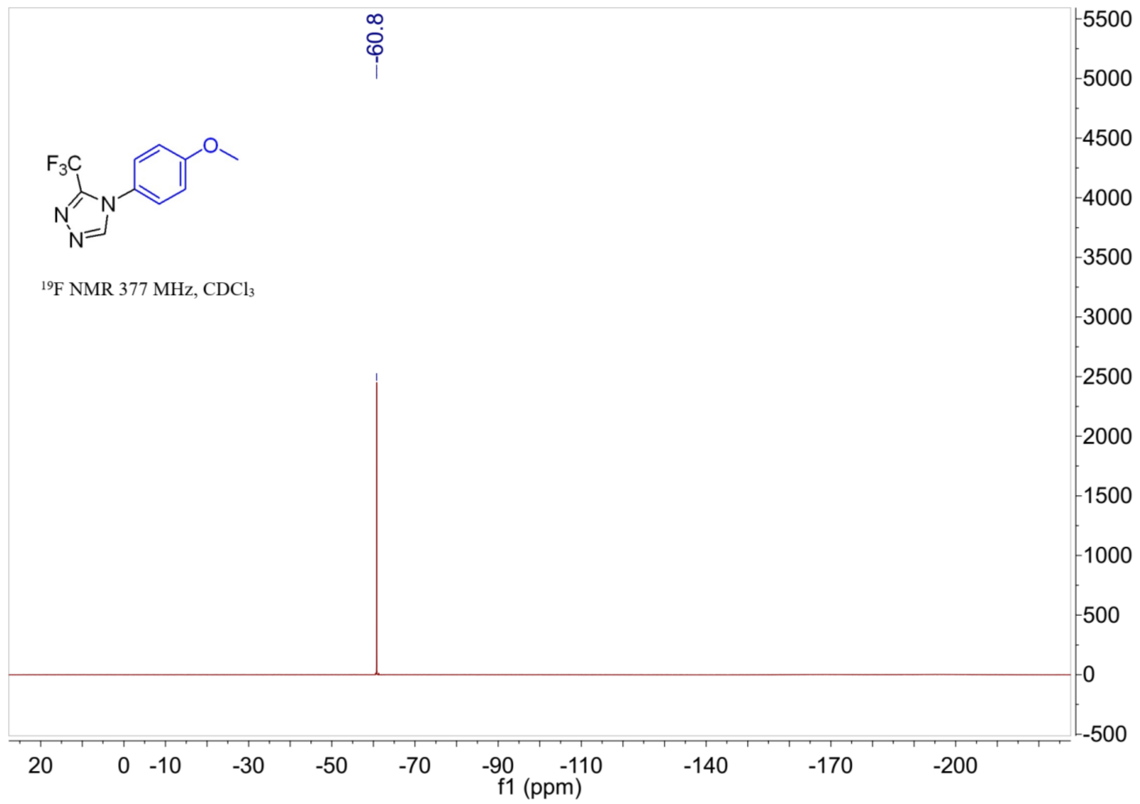

-60.8
F3C
N
N
N
O
19F NMR 377 MHz, CDCl3
5500
5000
4500
4000
3500
3000
2500
2000
1500
1000
500
0
-500
20
0
-10
-30
-50
-70
-90
-110
-140
-170
-200
f1 (ppm)

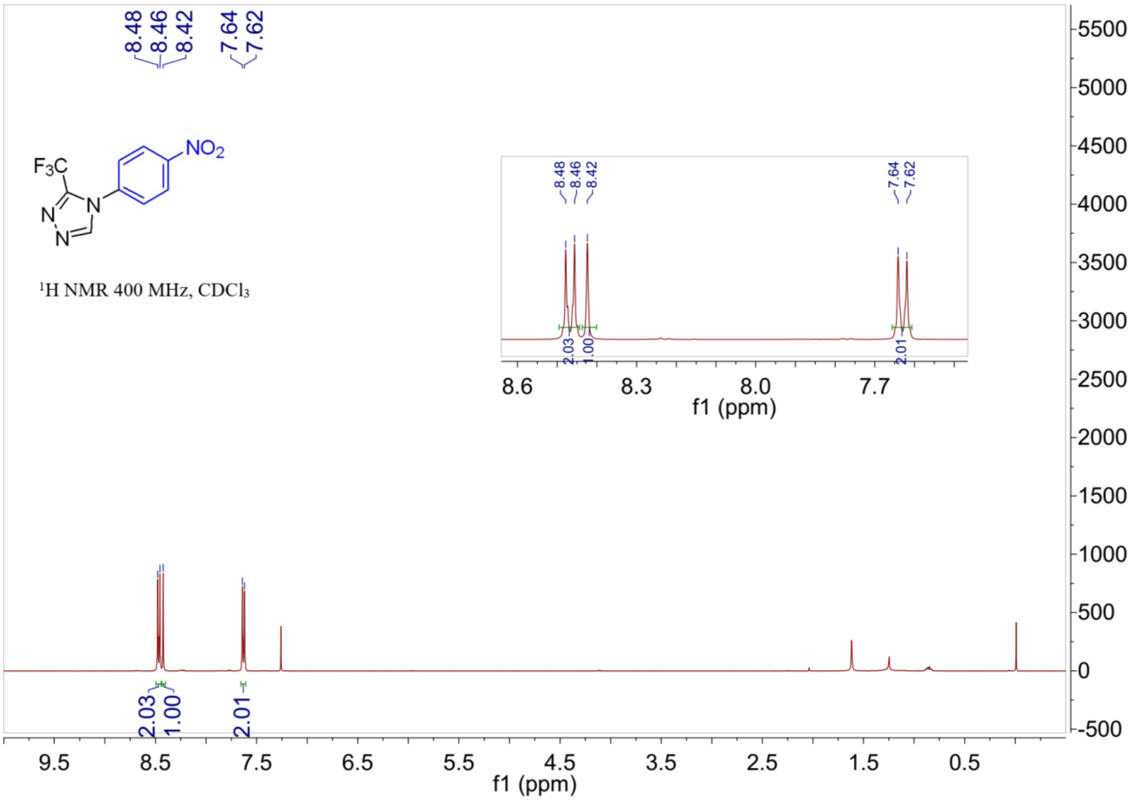
8.48
8.46
8.42
7.64
7.62
F3C
NO2
N
N
N
1H NMR 400 MHz, CDCl3
2.03
1.00
2.01
f1 (ppm)

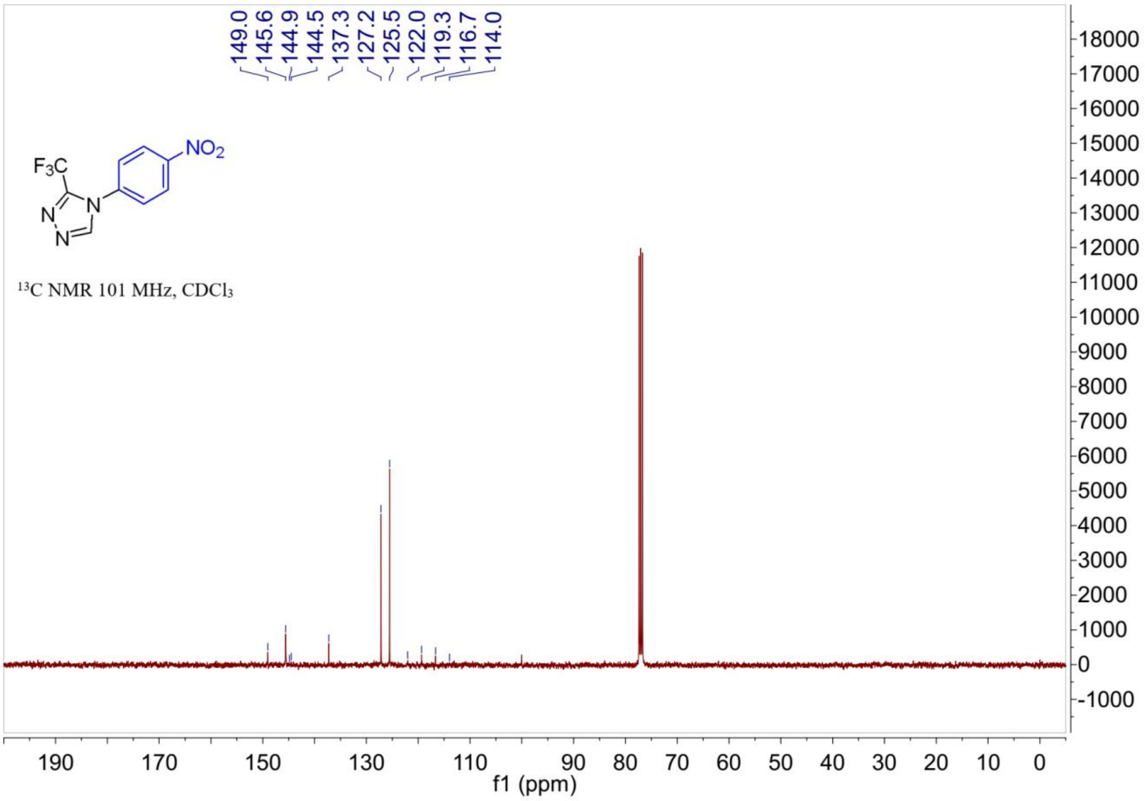
149.0
145.6
144.9
144.5
137.3
127.2
125.5
122.0
119.3
116.7
114.0
F3C
NO2
13C NMR 101 MHz, CDCl3
f1 (ppm)

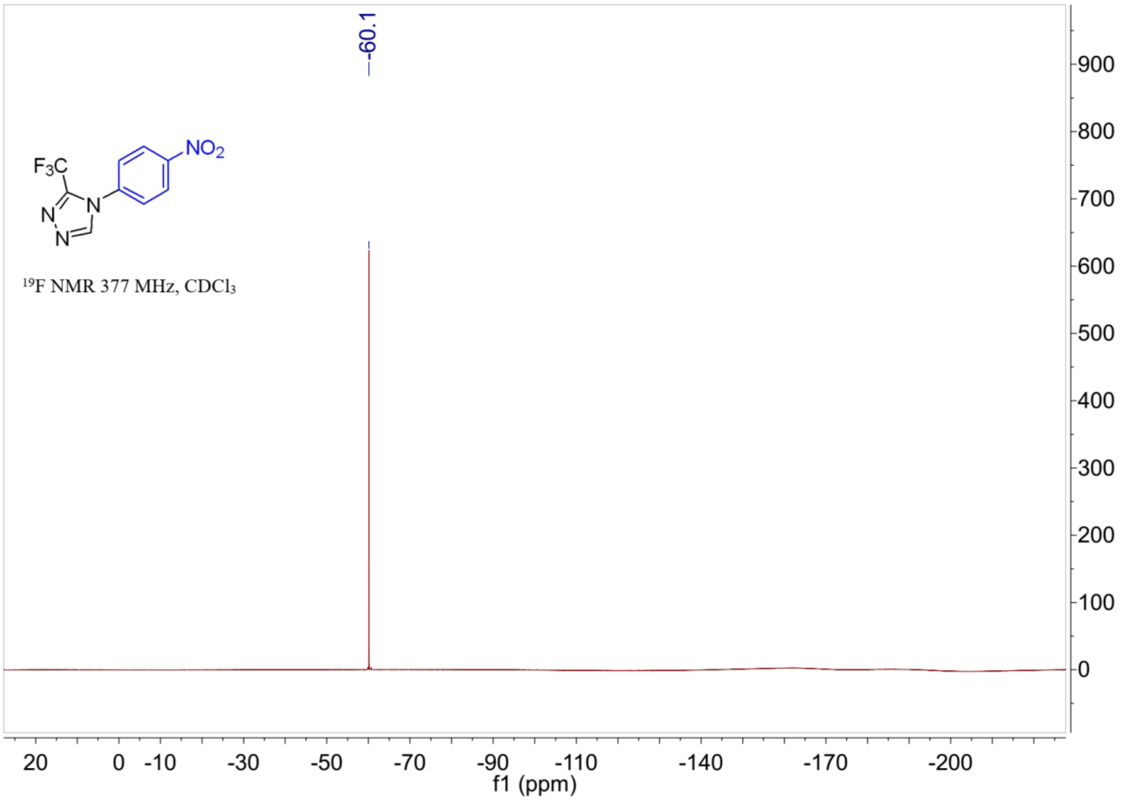
-60.1
F_3C
N
N
N
NO_2
^{19}F NMR 377 MHz, $CDCl_3$
900
800
700
600
500
400
300
200
100
0
20
0
-10
-30
-50
-70
-90
-110
-140
-170
-200
f1 (ppm)

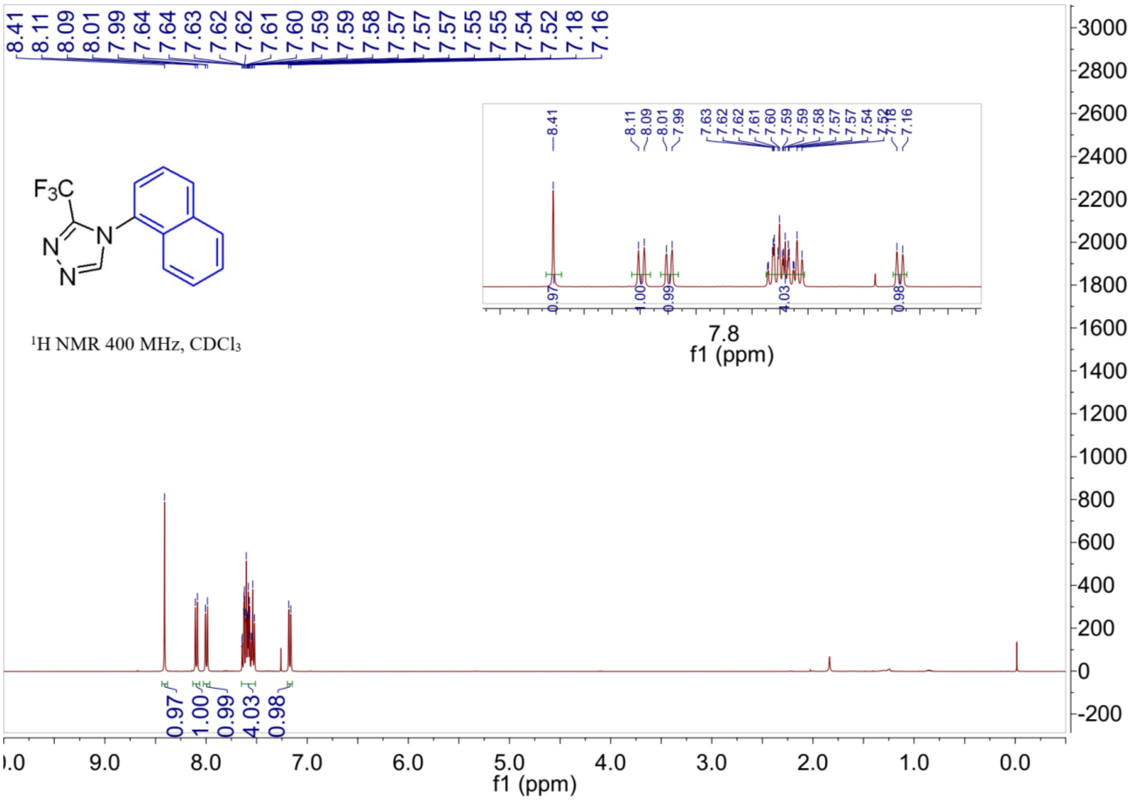
1H NMR 400 MHz, CDCl3

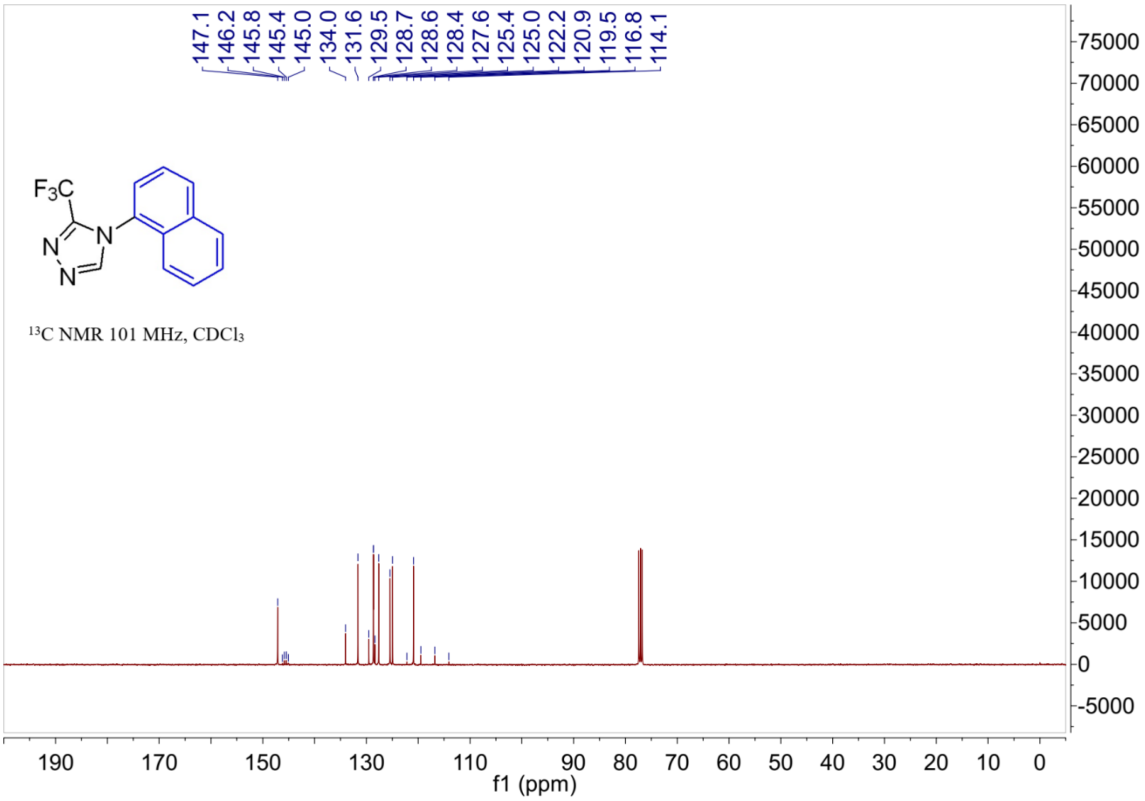
13C NMR 101 MHz, CDCl3

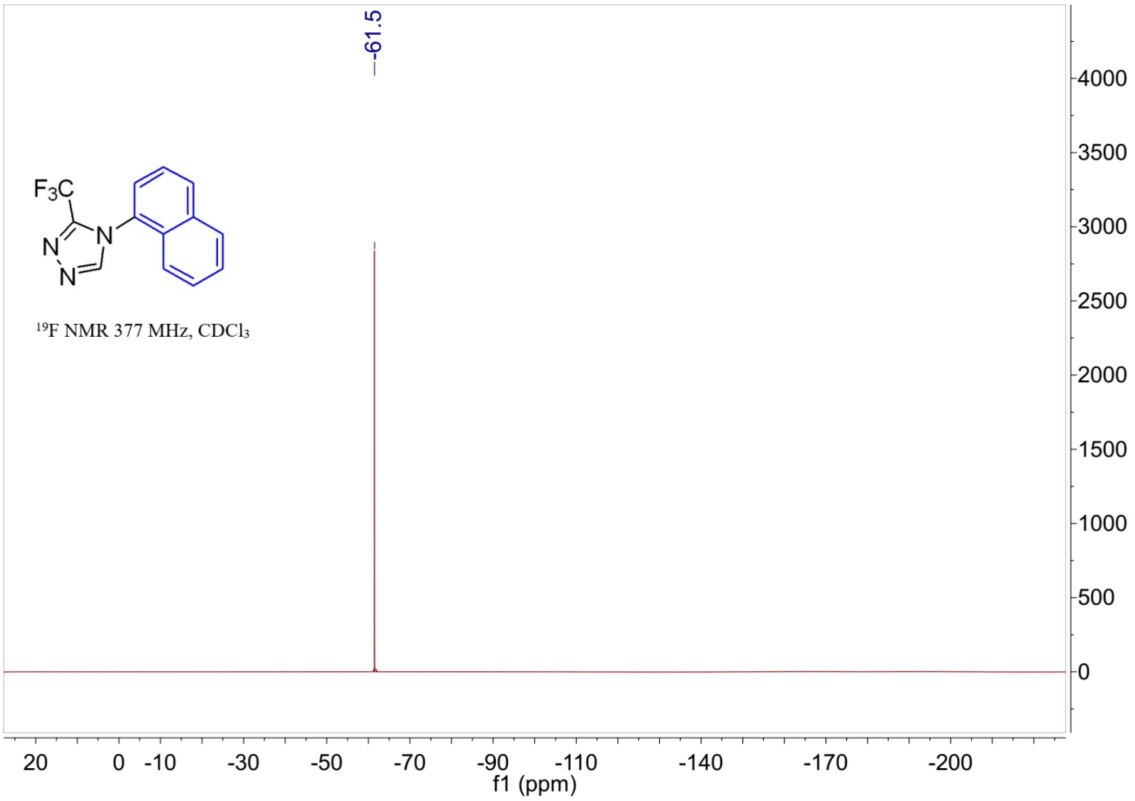
-61.5
F3C
N
N
N
19F NMR 377 MHz, CDCl3
4000
3500
3000
2500
2000
1500
1000
500
0
20
0
-10
-30
-50
-70
-90
-110
-140
-170
-200
f1 (ppm)

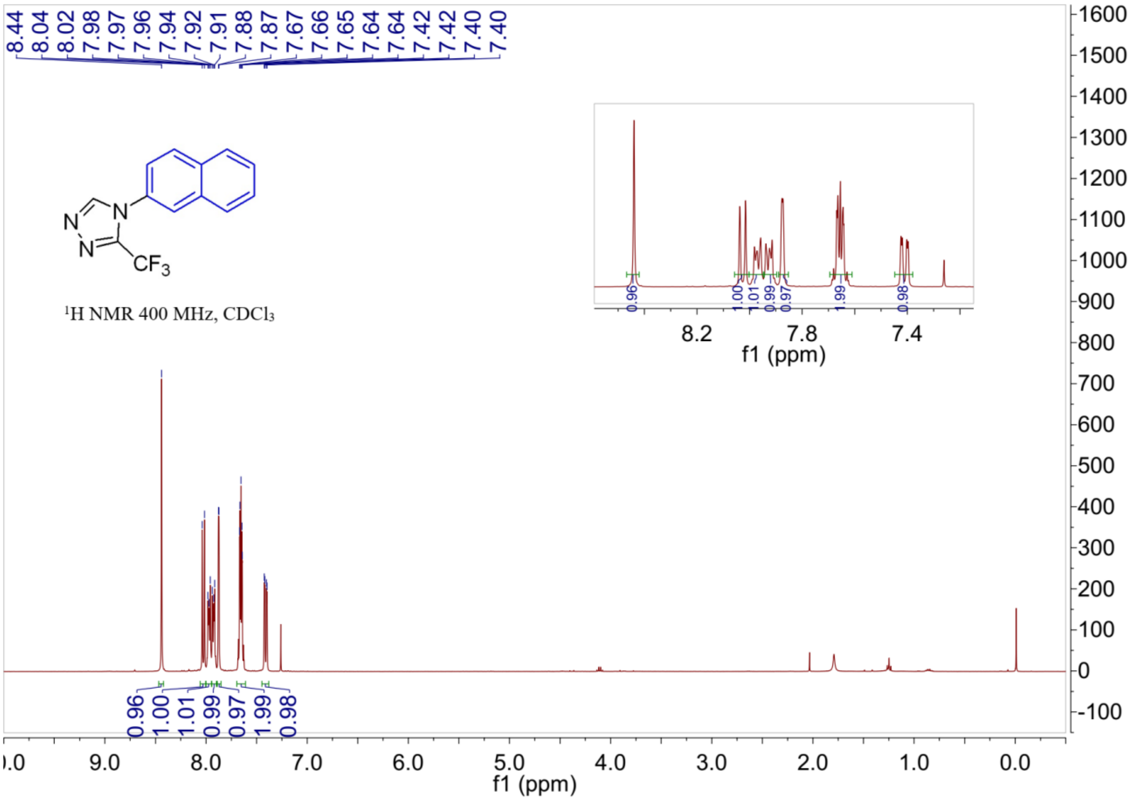
8.44
8.04
8.02
7.98
7.97
7.96
7.94
7.92
7.91
7.88
7.87
7.67
7.66
7.65
7.64
7.64
7.42
7.42
7.40
7.40
N
N
N
CF3
1H NMR 400 MHz, CDCl3
f1 (ppm)

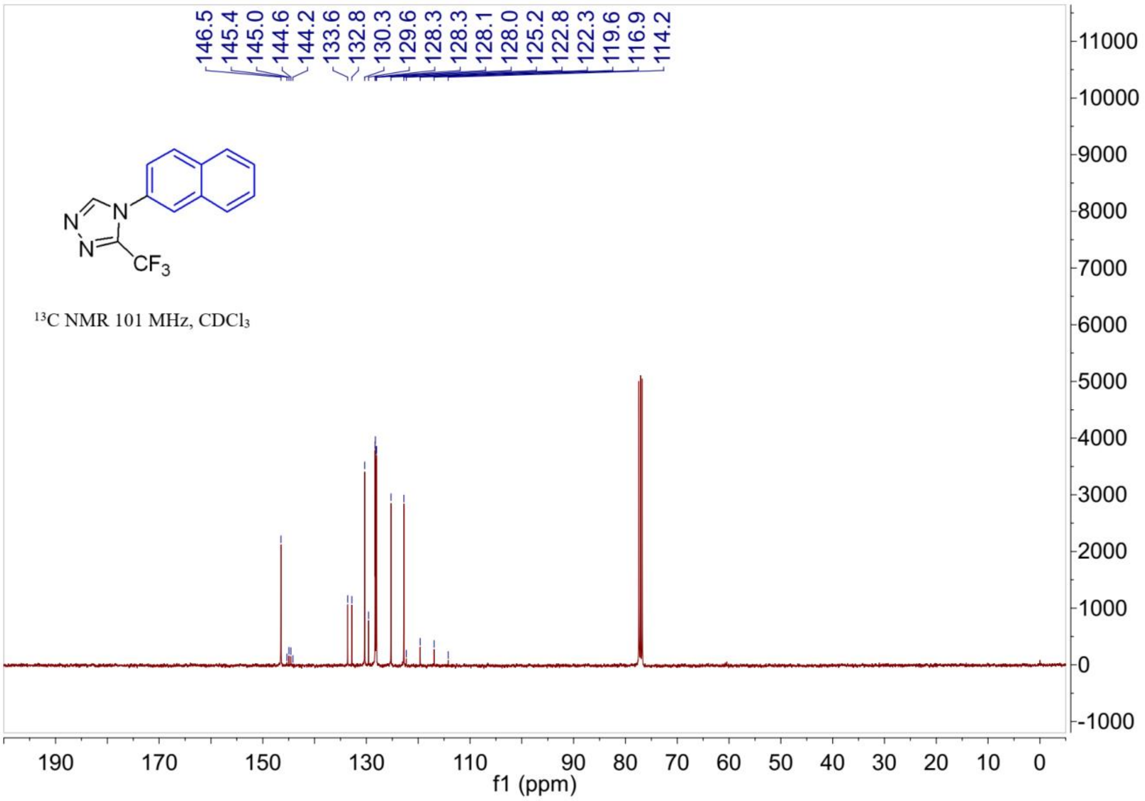
146.5
145.4
145.0
144.6
144.2
133.6
132.8
130.3
129.6
128.3
128.3
128.1
128.0
125.2
122.8
122.3
119.6
116.9
114.2
N
N
N
CF3
13C NMR 101 MHz, CDCl3
f1 (ppm)

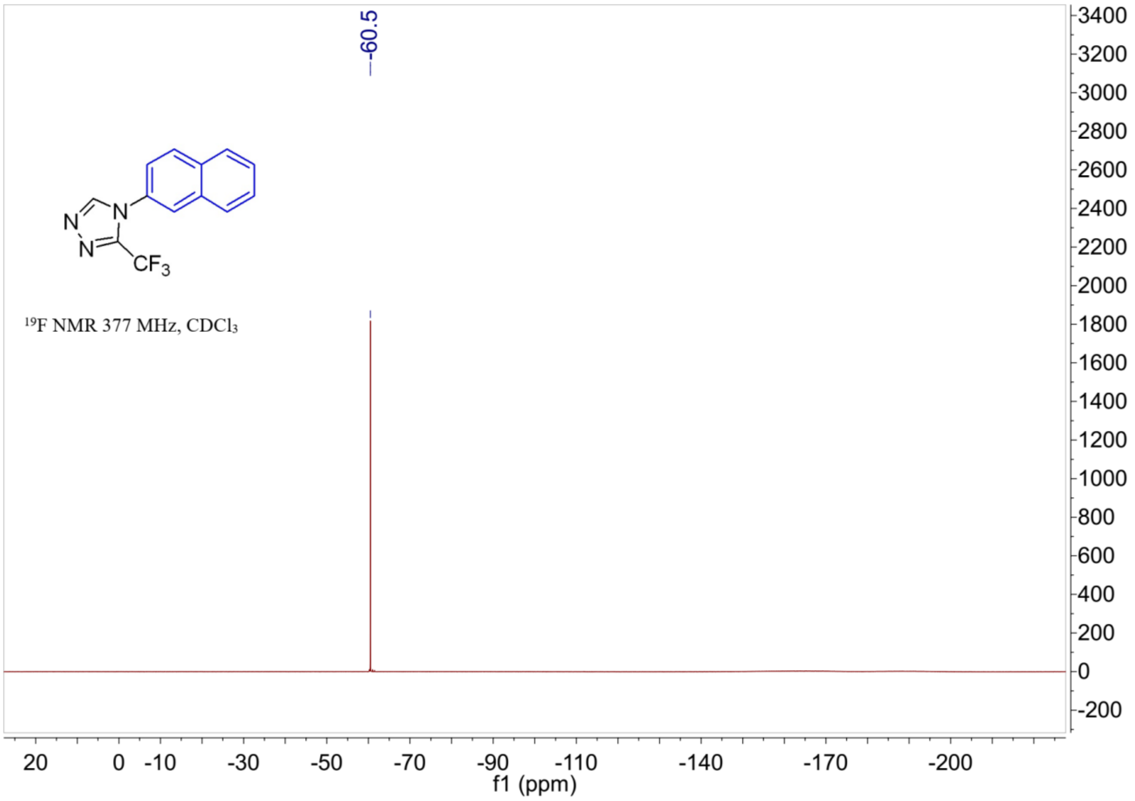

-60.5
N
N
N
CF3
19F NMR 377 MHz, CDCl3
3400
3200
3000
2800
2600
2400
2200
2000
1800
1600
1400
1200
1000
800
600
400
200
0
-200
20
0
-10
-30
-50
-70
-90
-110
-140
-170
-200
f1 (ppm)

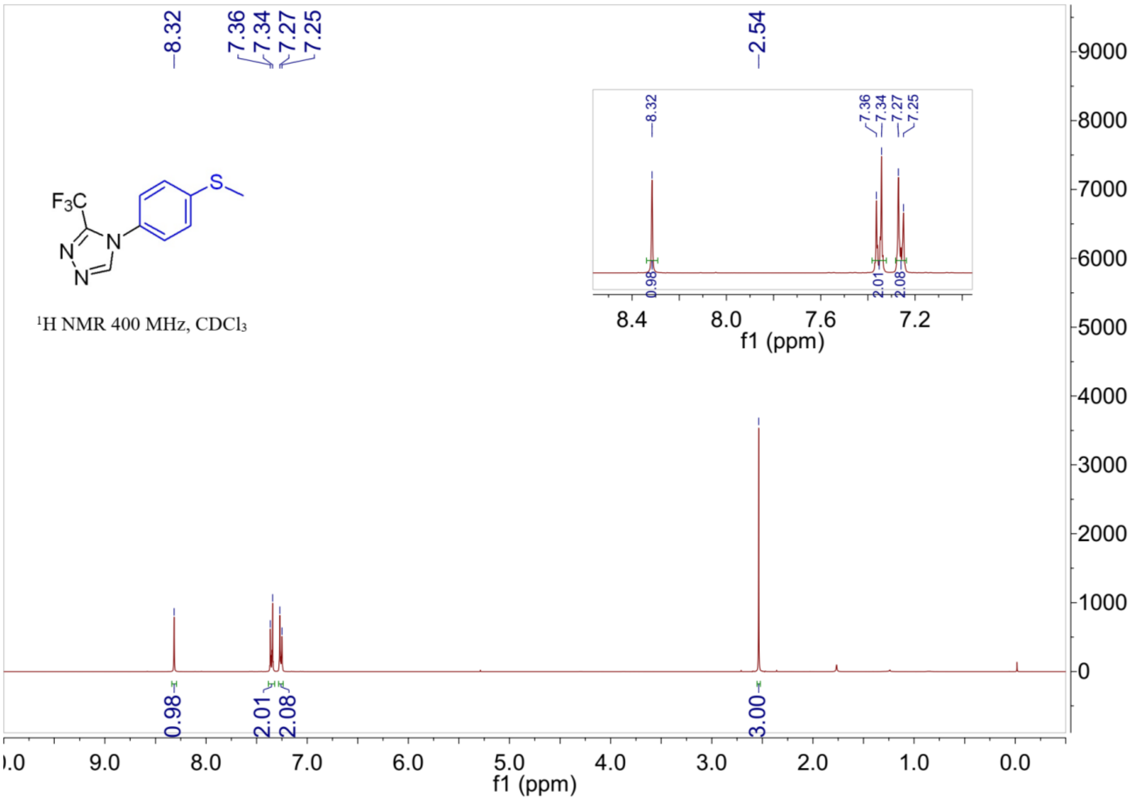

-8.32
7.36
7.34
7.27
7.25
-2.54
F_3C
S
N
N
N
1H NMR 400 MHz, CDCl3
0.98
2.01
2.08
3.00
f1 (ppm)

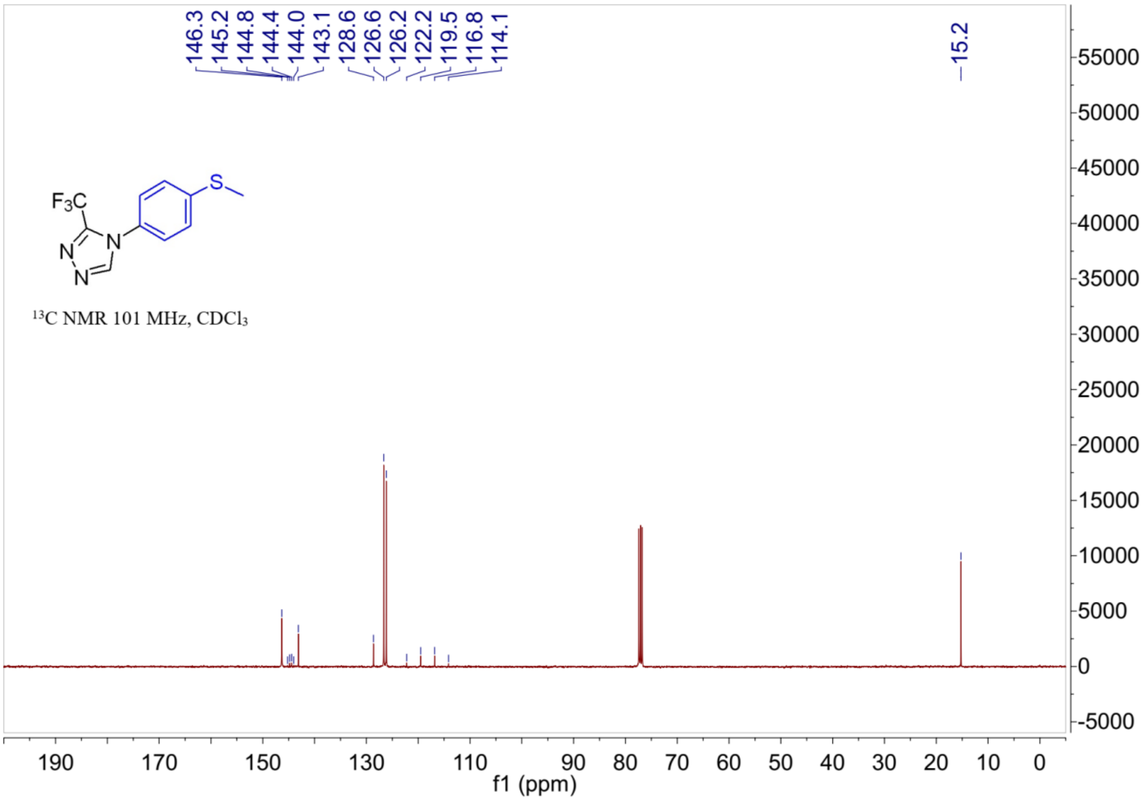

146.3
145.2
144.8
144.4
144.0
143.1
128.6
126.6
126.2
122.2
119.5
116.8
114.1
-15.2
F_3C
S
N
N
N
13C NMR 101 MHz, CDCl3
f1 (ppm)

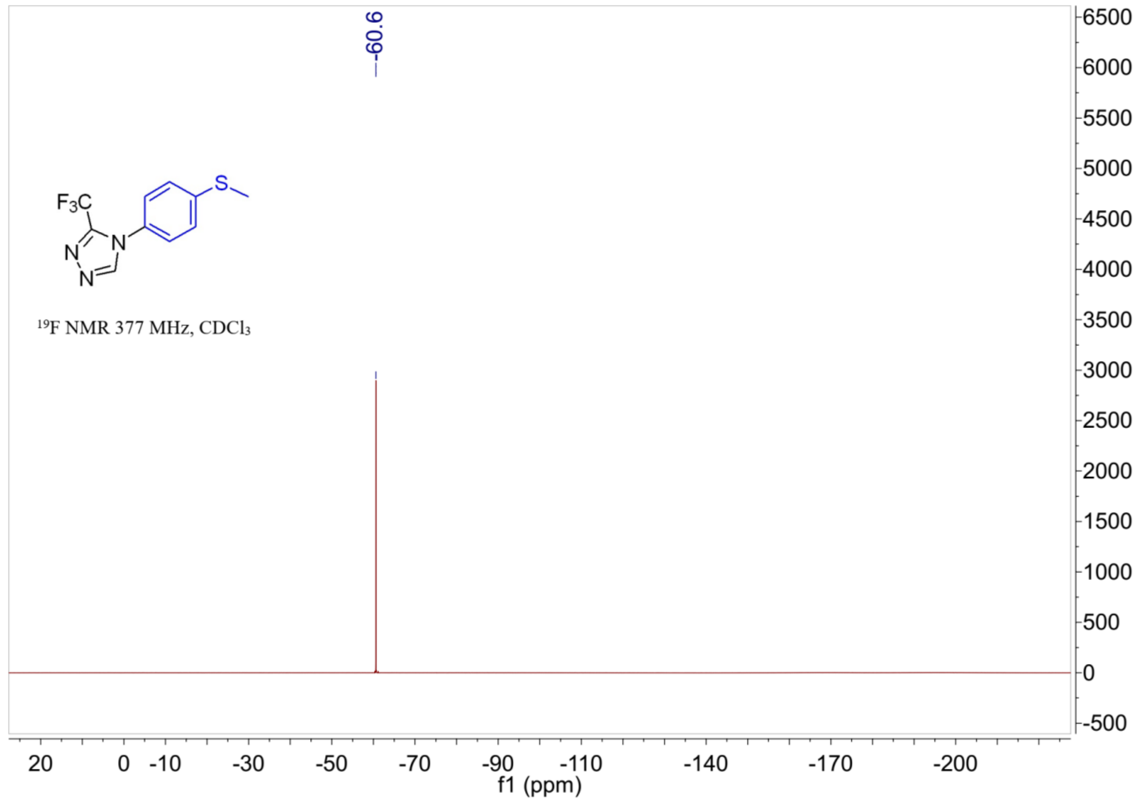
-60.6
F3C
N
N
N
S
19F NMR 377 MHz, CDCl3
6500
6000
5500
5000
4500
4000
3500
3000
2500
2000
1500
1000
500
0
-500
20
0
-10
-30
-50
-70
-90
-110
-140
-170
-200
f1 (ppm)

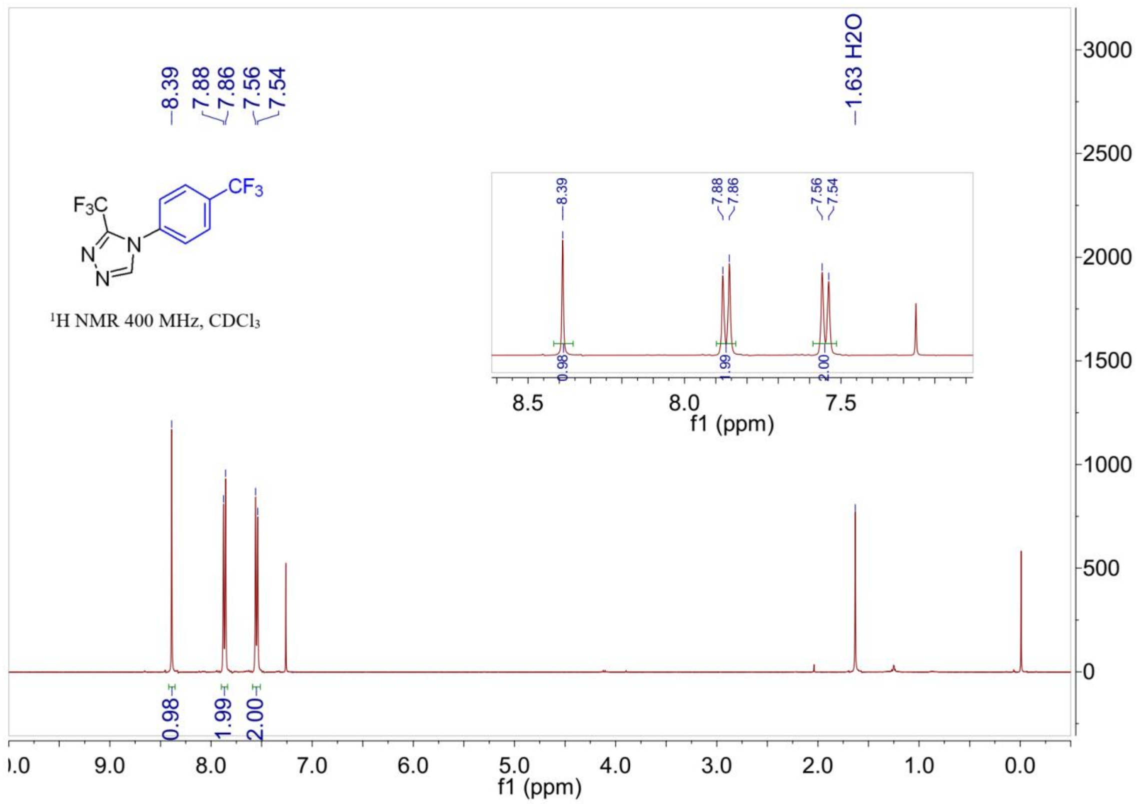
8.39
7.88
7.86
7.56
7.54
1.63 H2O
F3C
CF3
N
N
N
N
1H NMR 400 MHz, CDCl3
0.98
1.99
2.00
f1 (ppm)

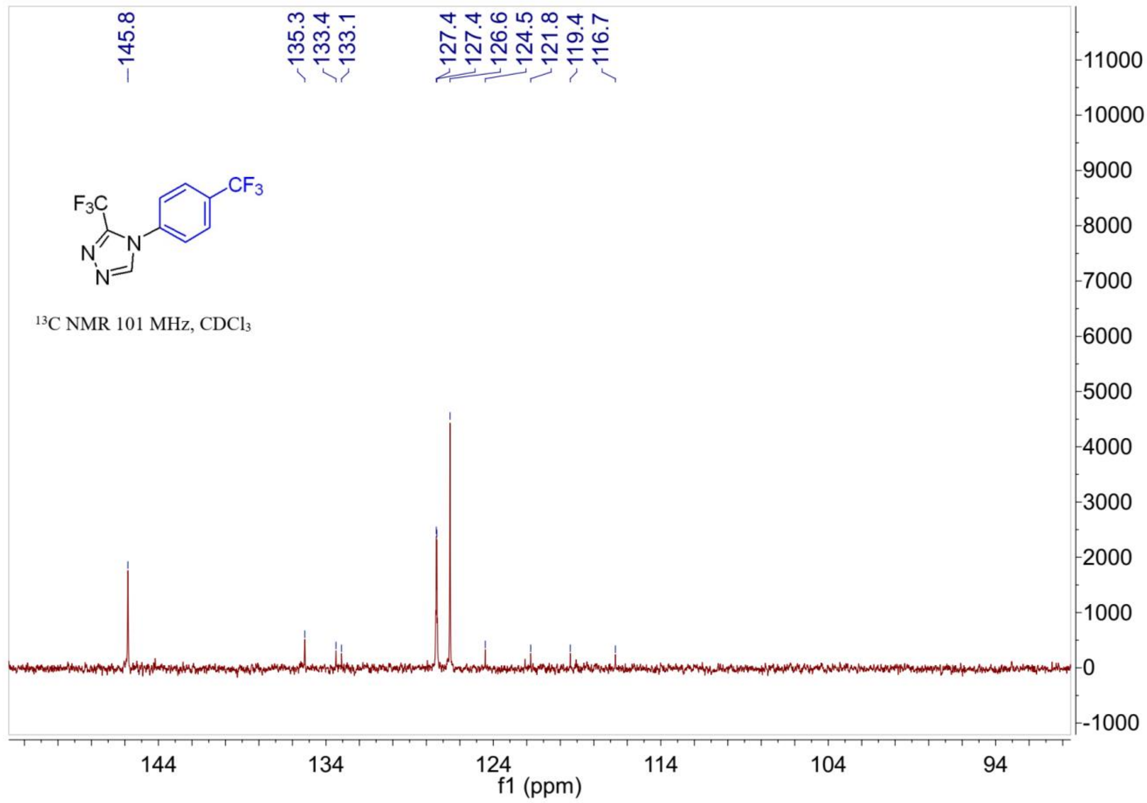
145.8
135.3
133.4
133.1
127.4
127.4
126.6
124.5
121.8
119.4
116.7
F3C
CF3
13C NMR 101 MHz, CDCl3
f1 (ppm)

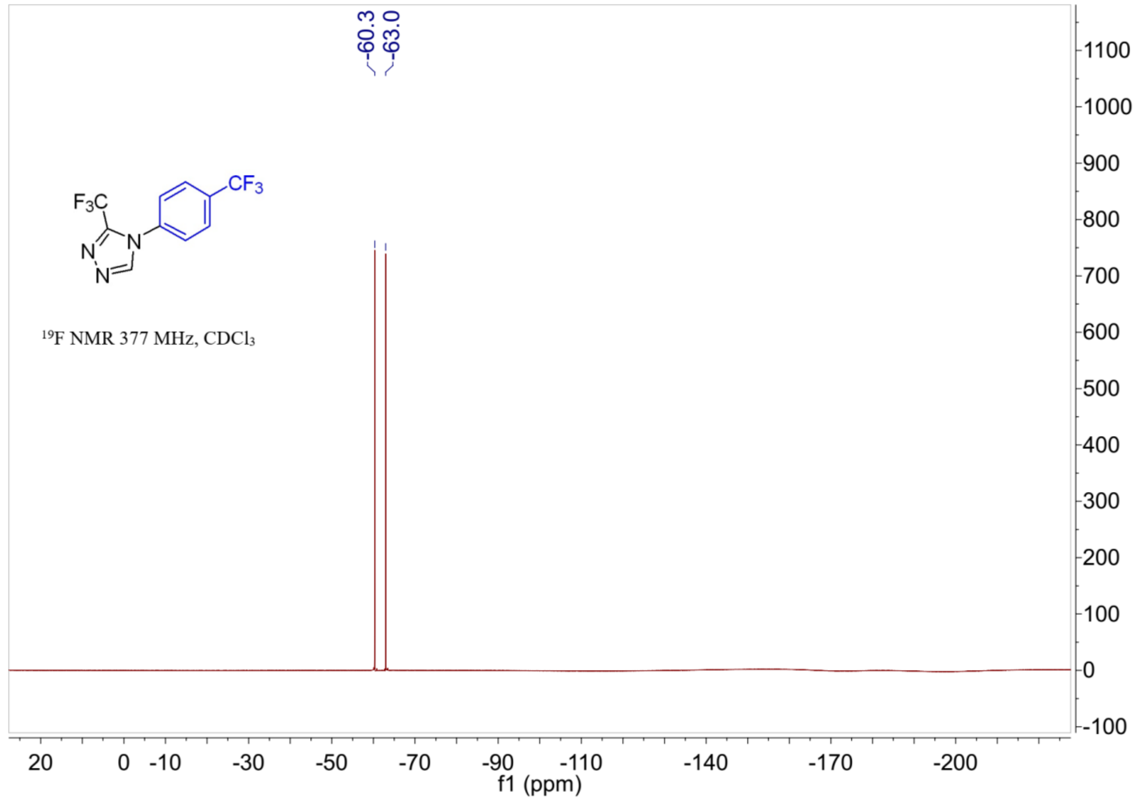

-60.3
-63.0
F3C
CF3
N
N
N
N
19F NMR 377 MHz, CDCl3
f1 (ppm)

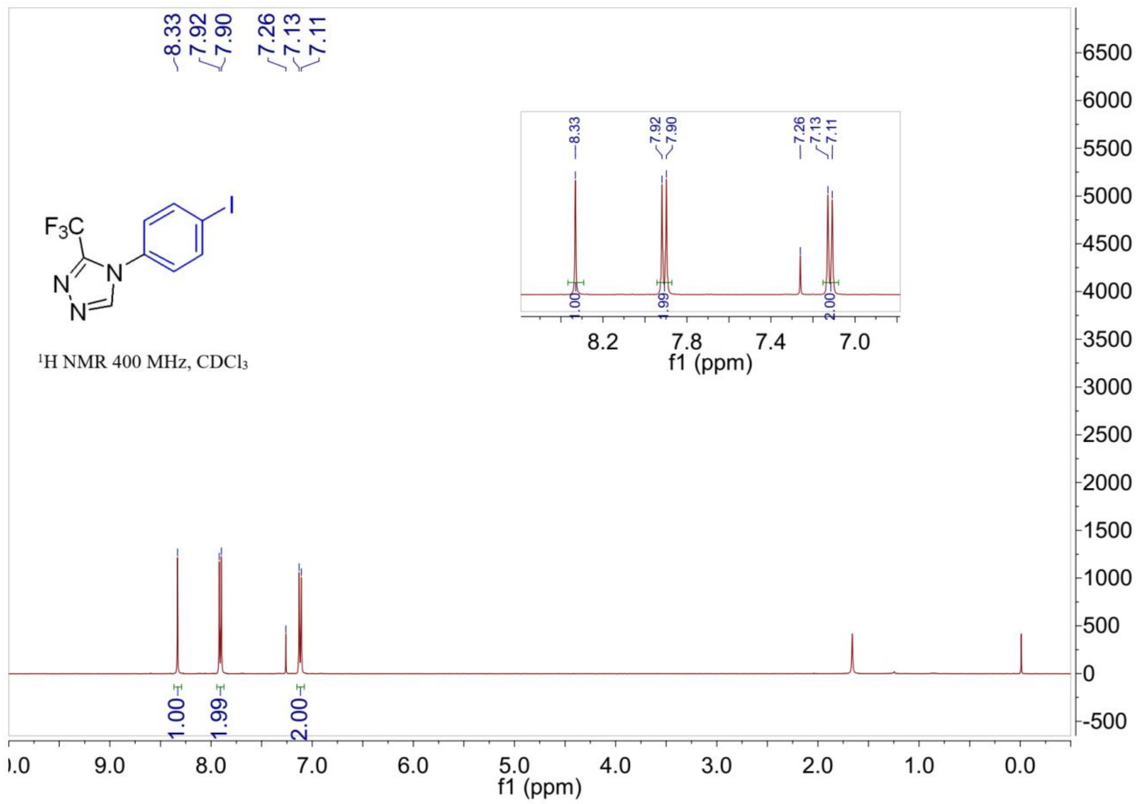

8.33
7.92
7.90
7.26
7.13
7.11
F_3C
N
N
N
I
1H NMR 400 MHz, CDCl3
1.00
1.99
2.00
f1 (ppm)

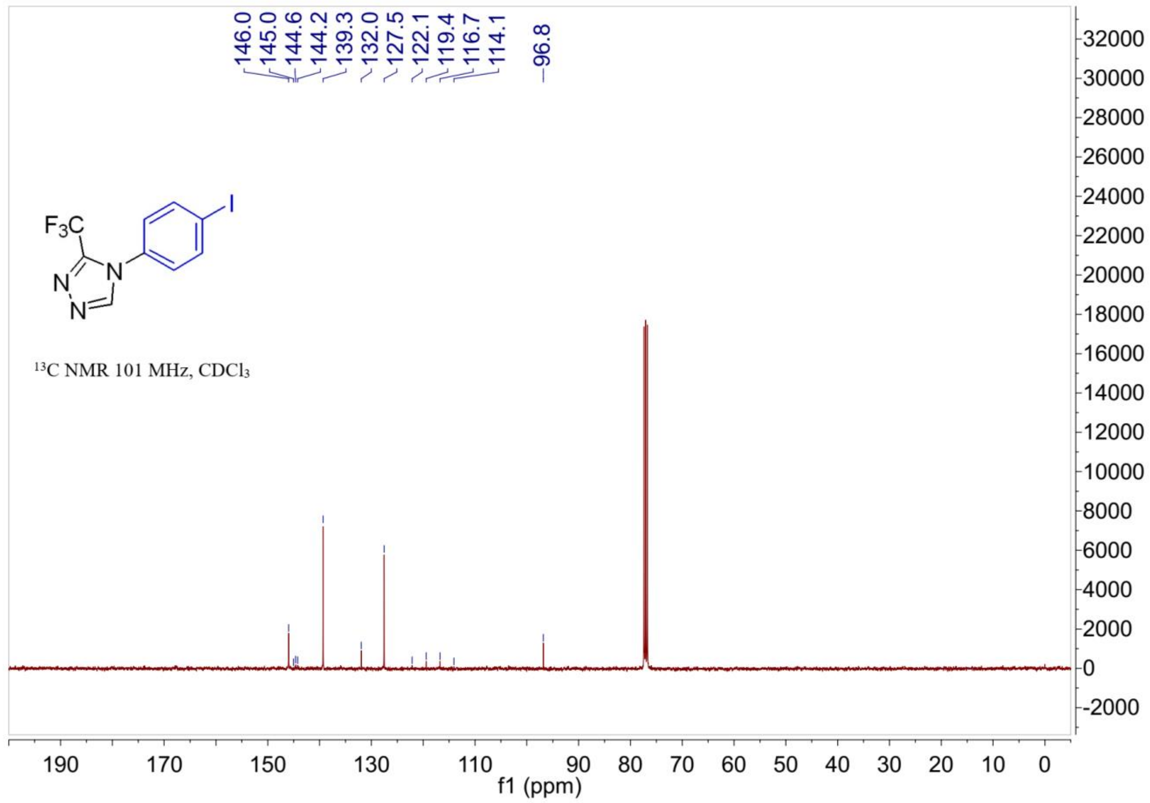

146.0
145.0
144.6
144.2
139.3
132.0
127.5
122.1
119.4
116.7
114.1
96.8
F_3C
N
N
N
I
13C NMR 101 MHz, CDCl3
f1 (ppm)

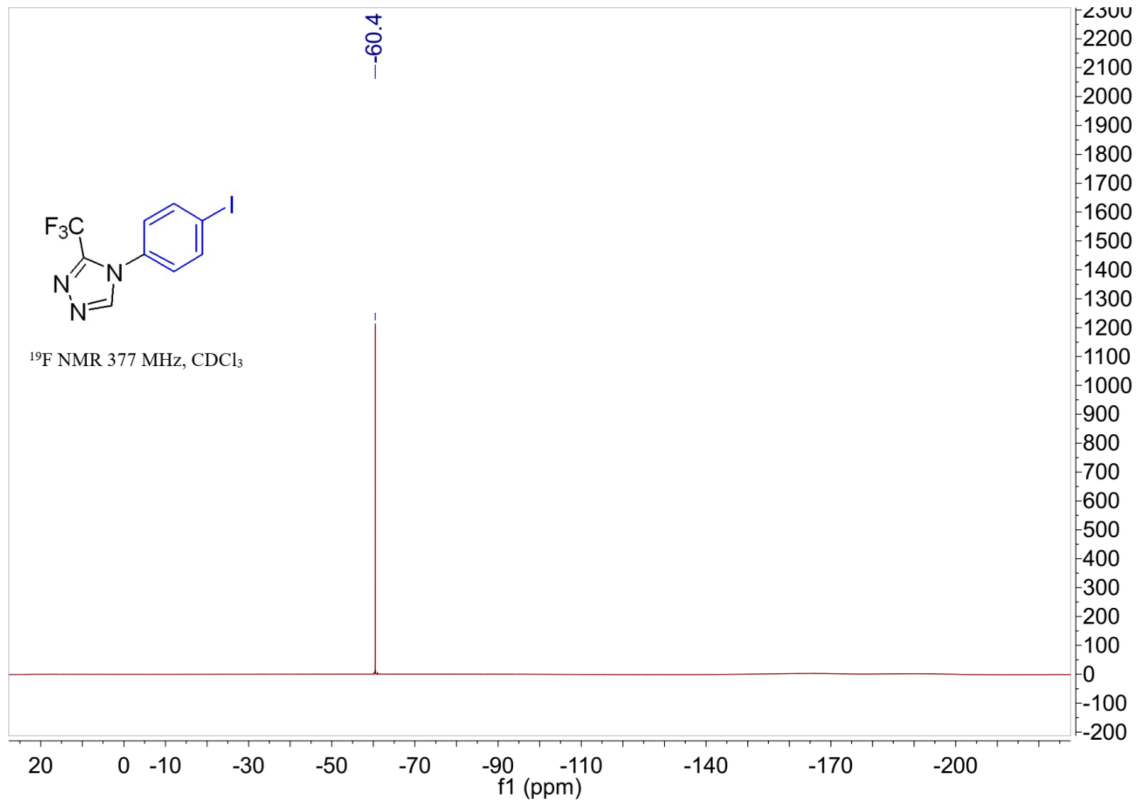
-60.4
F3C
N
N
N
I
19F NMR 377 MHz, CDCl3
f1 (ppm)

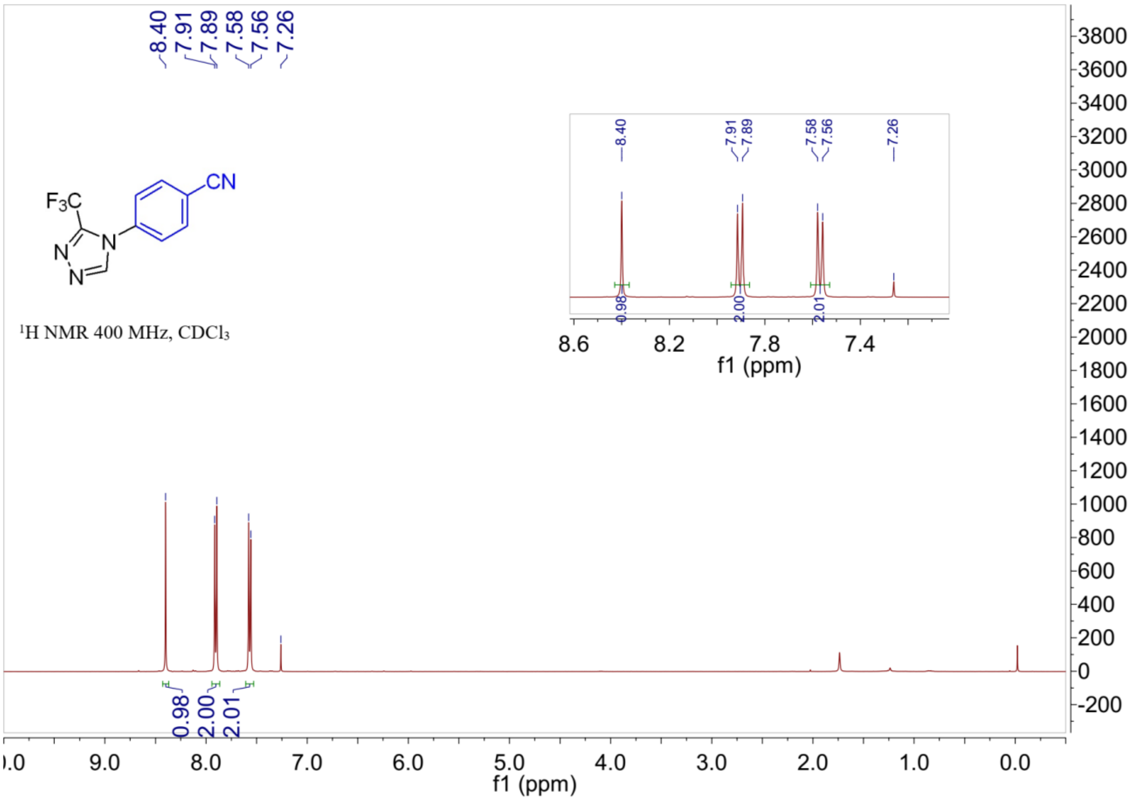
8.40
7.91
7.89
7.58
7.56
7.26
F3C
CN
N
N
N
1H NMR 400 MHz, CDCl3
0.98
2.00
2.01
f1 (ppm)

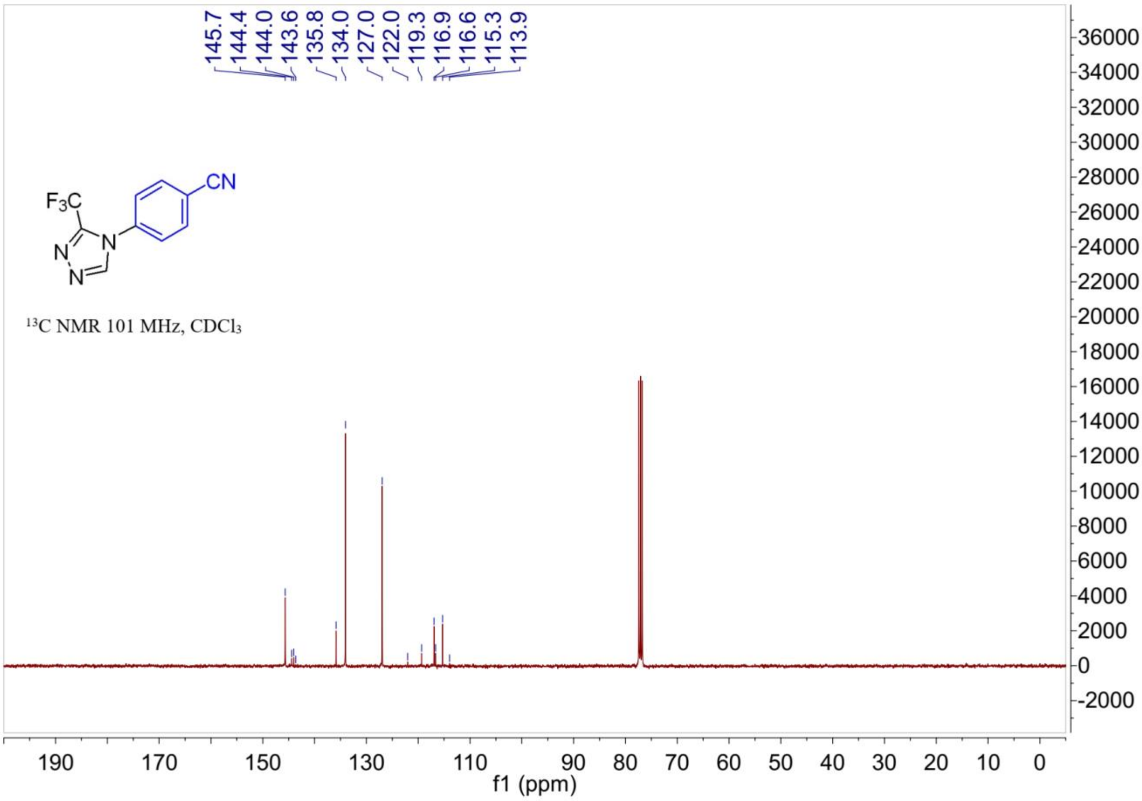
145.7
144.4
144.0
143.6
135.8
134.0
127.0
122.0
119.3
116.9
116.6
115.3
113.9
F3C
CN
N
N
N
13C NMR 101 MHz, CDCl3
f1 (ppm)

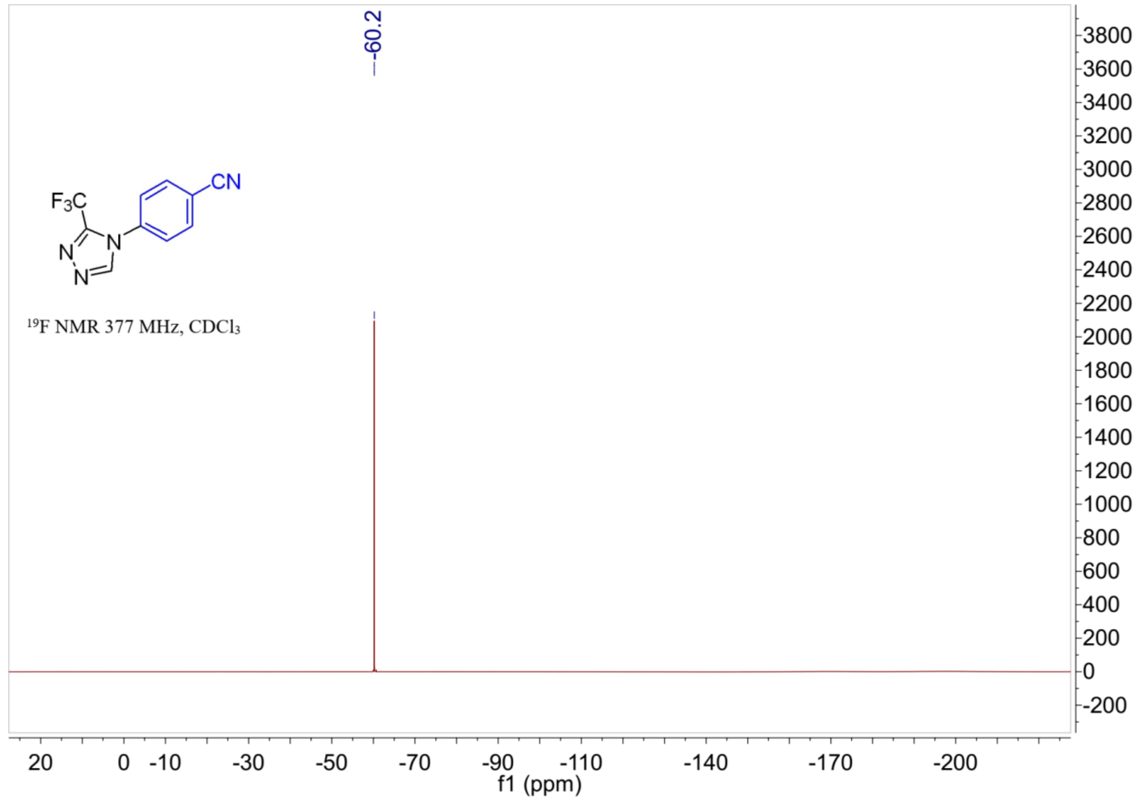
-60.2
F3C
N
N
N
CN
19F NMR 377 MHz, CDCl3
3800
3600
3400
3200
3000
2800
2600
2400
2200
2000
1800
1600
1400
1200
1000
800
600
400
200
0
-200
20
0
-10
-30
-50
-70
-90
-110
-140
-170
-200
f1 (ppm)

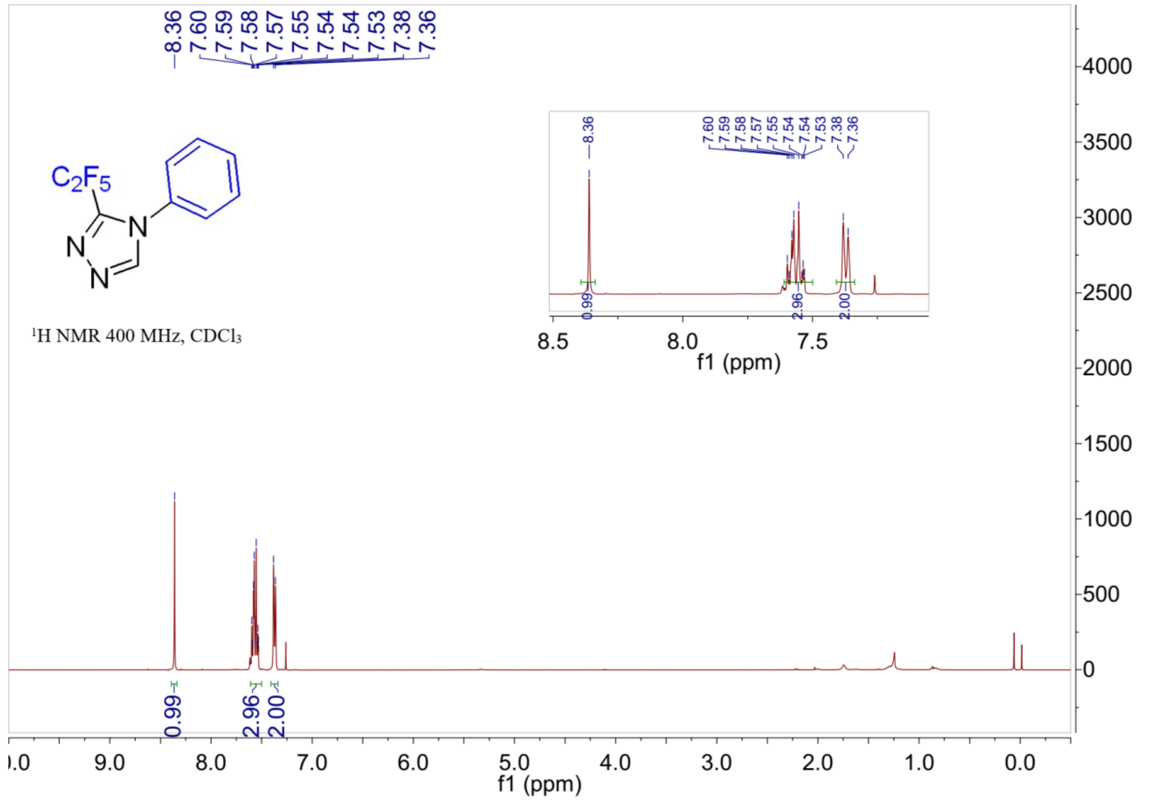
C2F5
N
N
N
1H NMR 400 MHz, CDCl3
f1 (ppm)

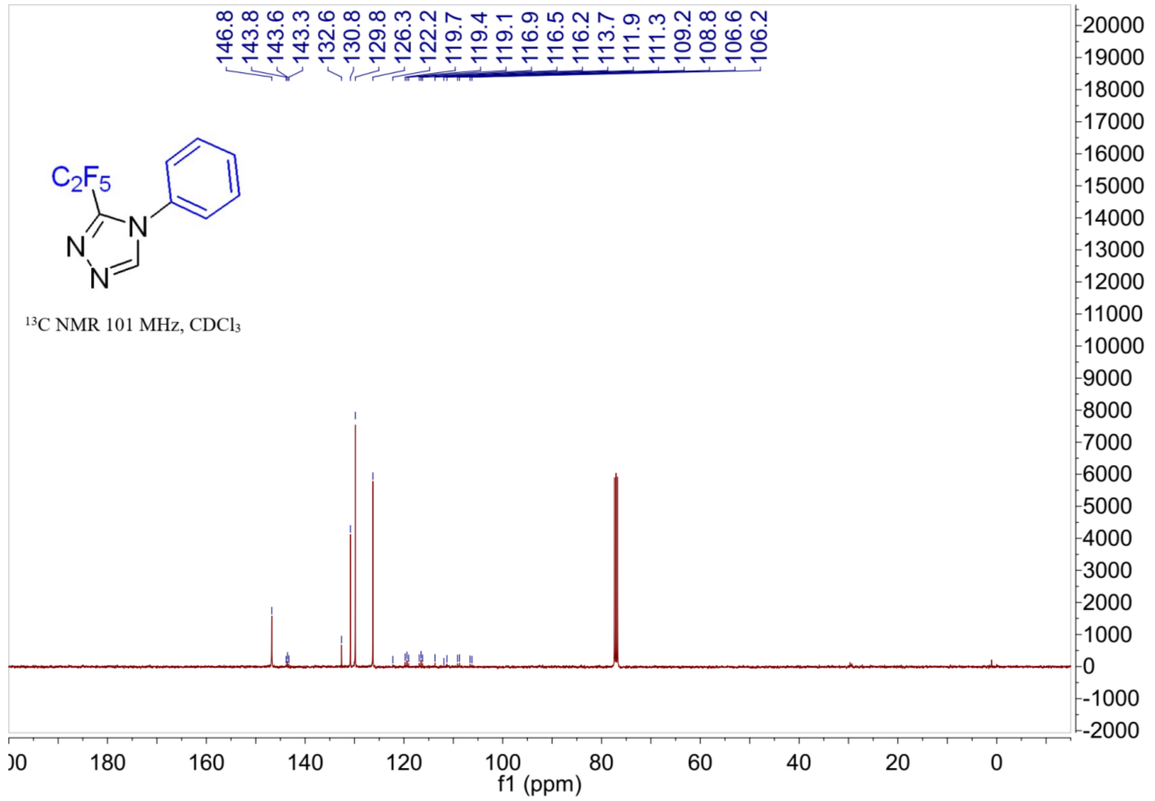
C2F5
N
N
N
13C NMR 101 MHz, CDCl3
f1 (ppm)

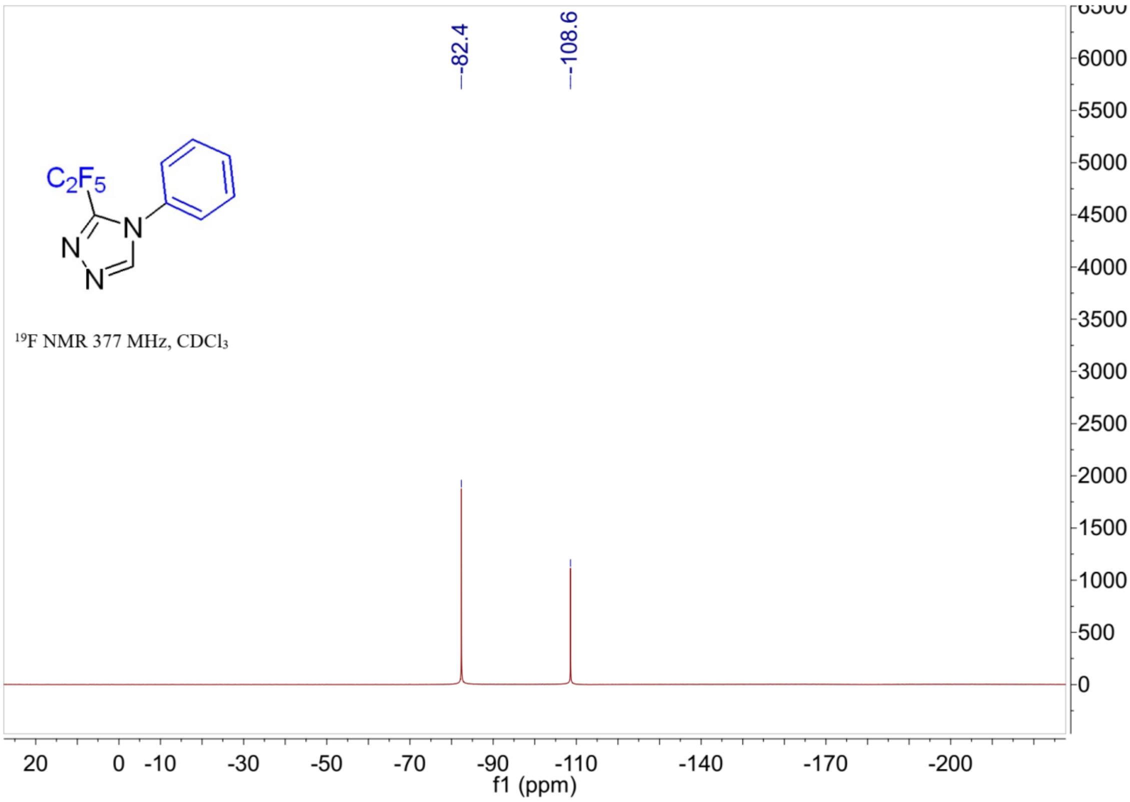

-82.4
-108.6
C_2F_5
N
N
N
^{19}F NMR 377 MHz, $CDCl_3$
6500
6000
5500
5000
4500
4000
3500
3000
2500
2000
1500
1000
500
0
20
0
-10
-30
-50
-70
-90
-110
-140
-170
-200
f1 (ppm)

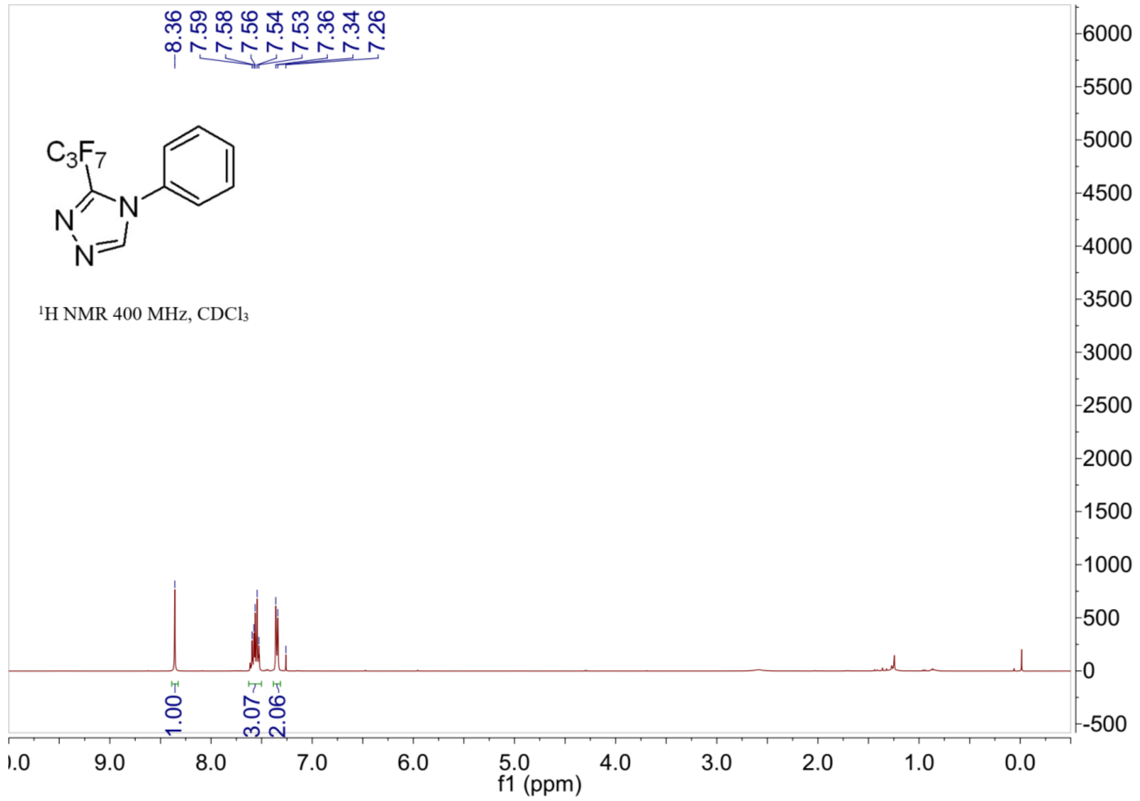

8.36
7.59
7.58
7.56
7.54
7.53
7.36
7.34
7.26
C3F7
N
N
N
1H NMR 400 MHz, CDCl3
1.00
3.07
2.06
f1 (ppm)

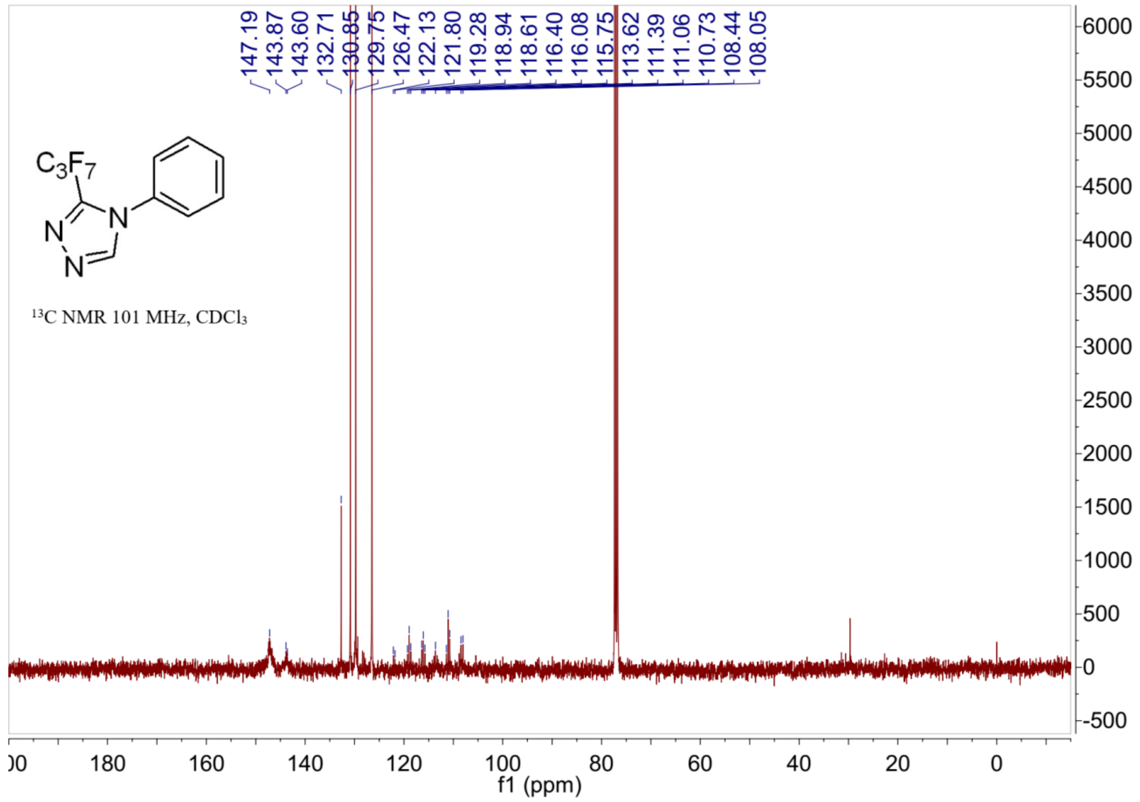

147.19
143.87
143.60
132.71
130.85
129.75
126.47
122.13
121.80
119.28
118.94
118.61
116.40
116.08
115.75
113.62
111.39
111.06
110.73
108.44
108.05
C3F7
N
N
N
13C NMR 101 MHz, CDCl3
f1 (ppm)

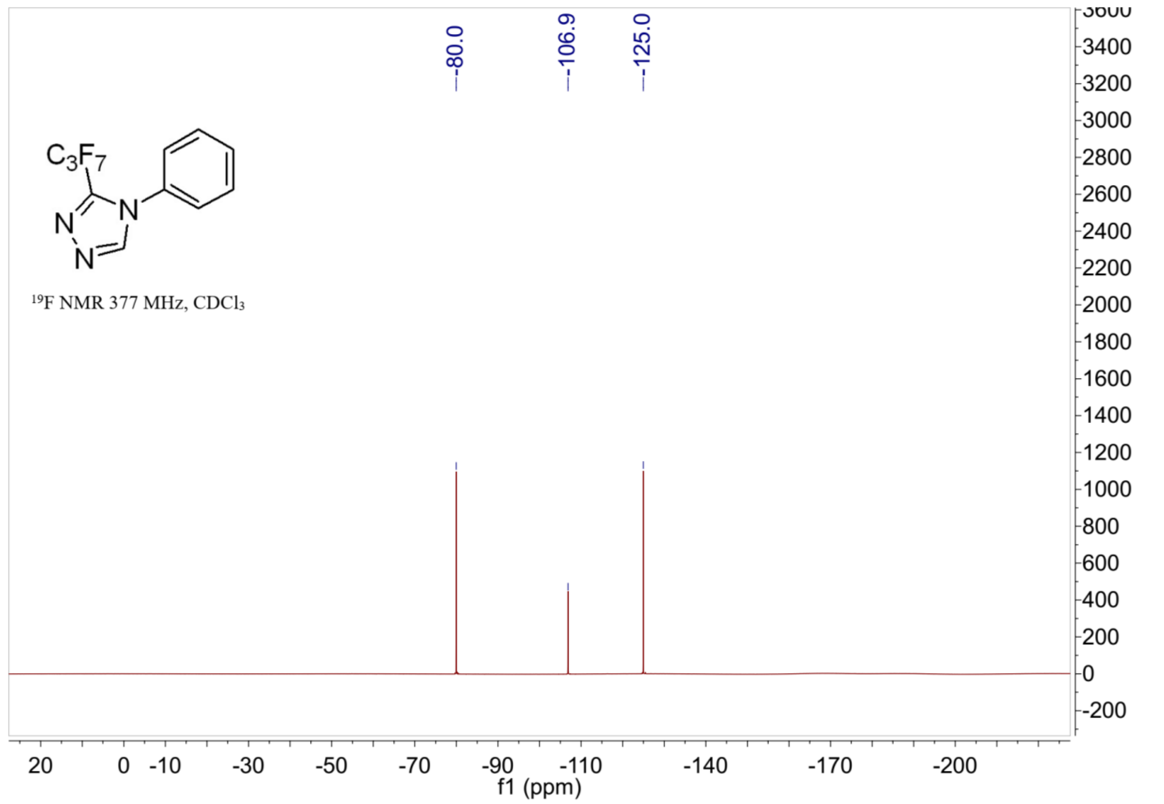

-80.0
-106.9
-125.0
C_3F_7
N
N
N
^{19}F NMR 377 MHz, $CDCl_3$
f1 (ppm)

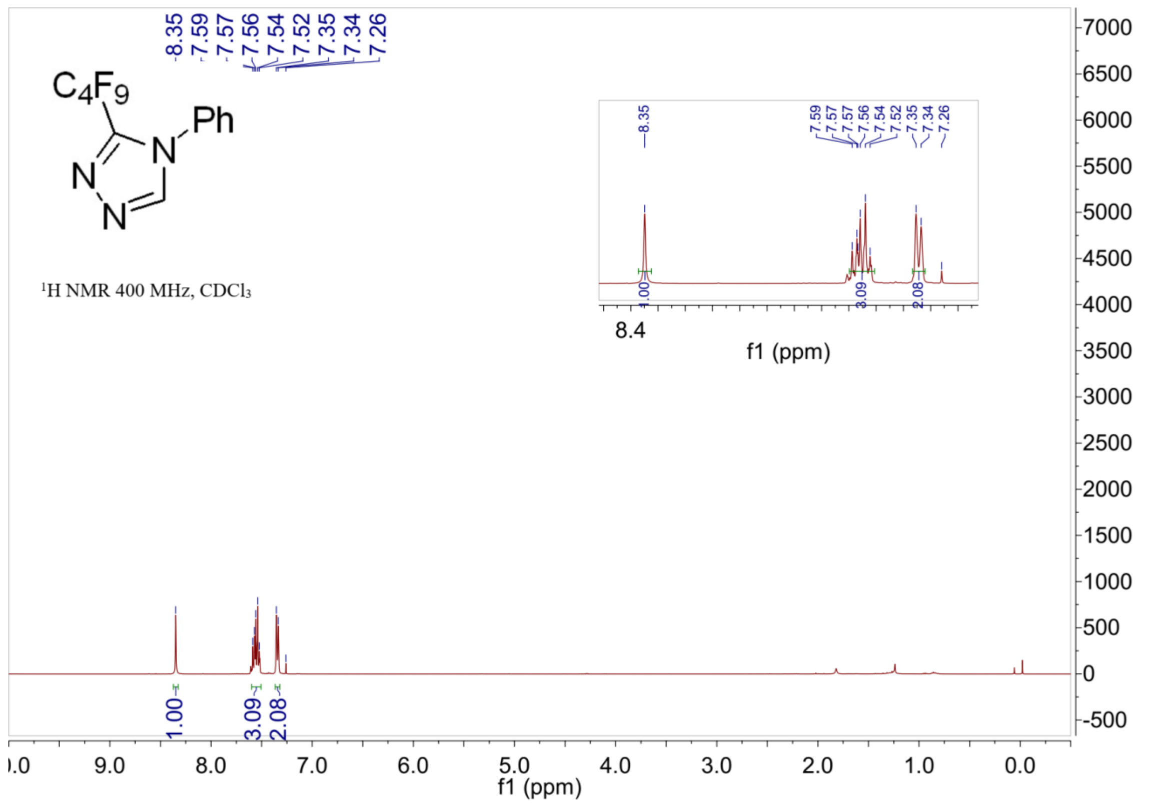

-8.35
7.59
7.57
7.56
7.54
7.52
7.35
7.34
7.26
C4F9
N
Ph
N
N
1H NMR 400 MHz, CDCl3
1.00
3.09
2.08
f1 (ppm)

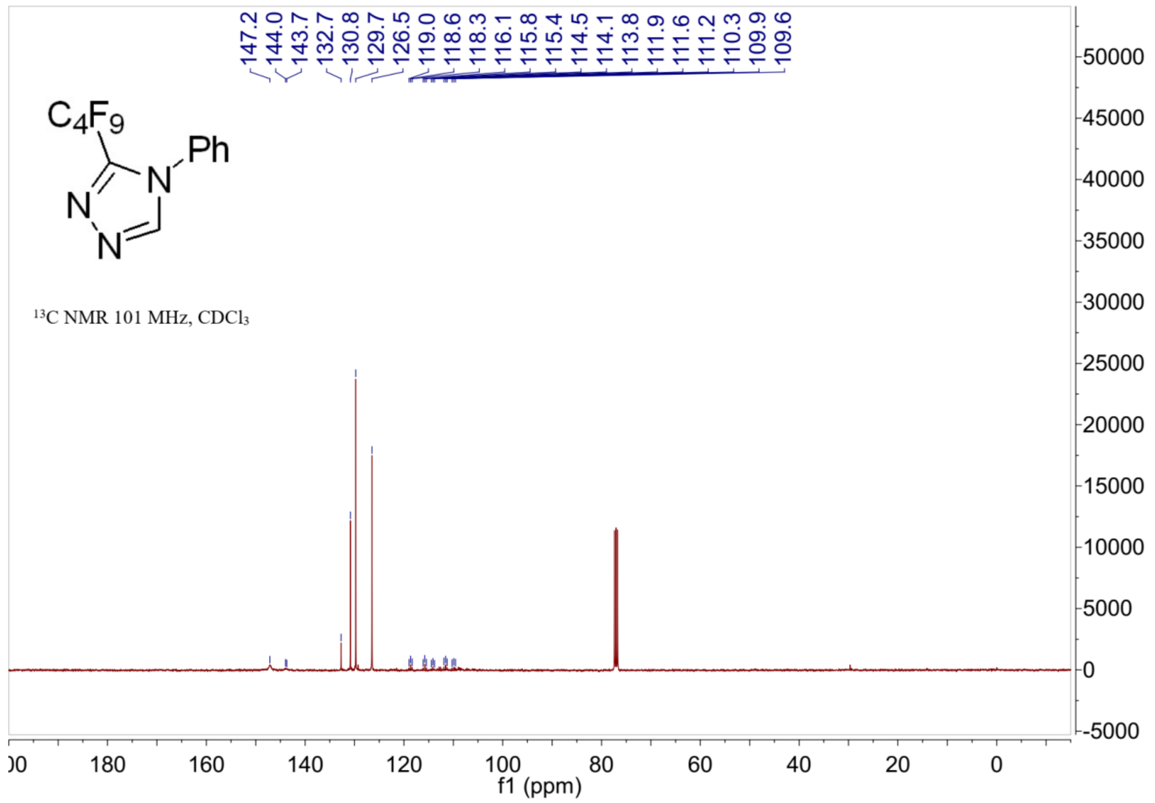

147.2
144.0
143.7
132.7
130.8
129.7
126.5
119.0
118.6
118.3
116.1
115.8
115.4
114.5
114.1
113.8
111.9
111.6
111.2
110.3
109.9
109.6
C4F9
N
Ph
N
N
13C NMR 101 MHz, CDCl3
f1 (ppm)

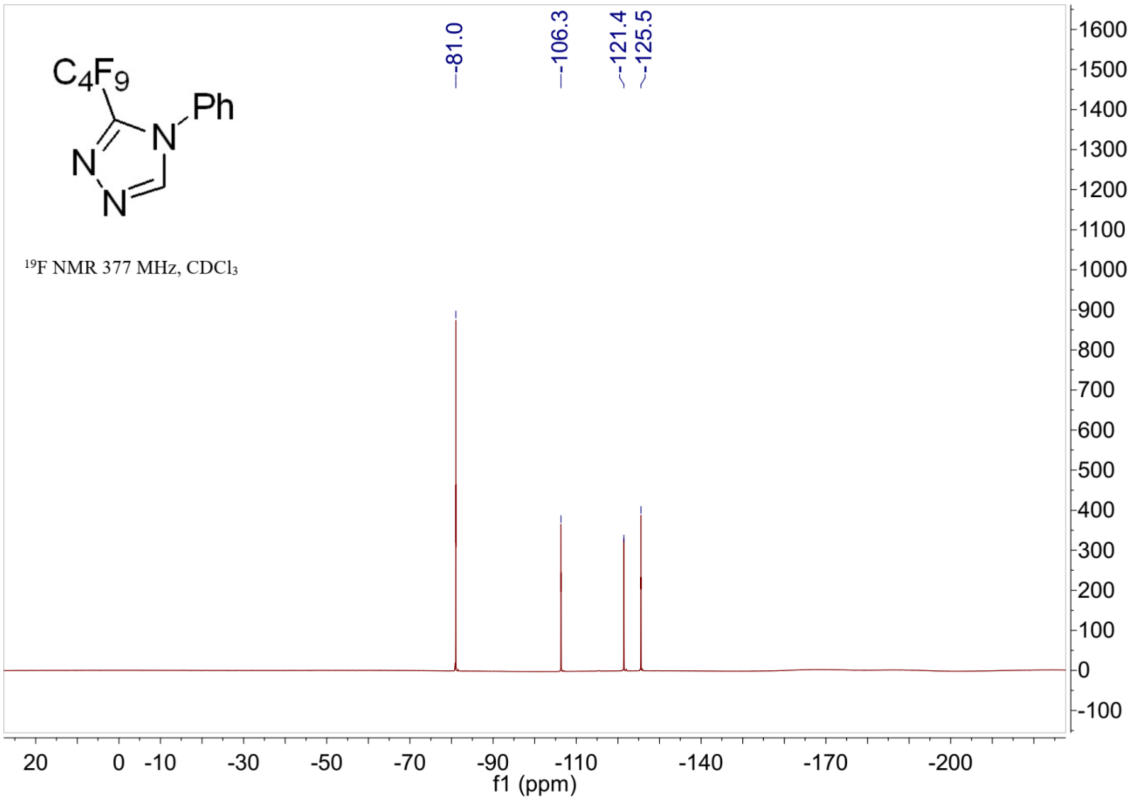

C4F9
N
Ph
N
N
19F NMR 377 MHz, CDCl3
-81.0
-106.3
-121.4
-125.5
f1 (ppm)
